# Habitat deterioration promotes the evolution of direct development in metamorphosing species

**DOI:** 10.1111/evo.14040

**Published:** 2020-06-24

**Authors:** Hanna ten Brink, Renske E. Onstein, André M. de Roos

**Affiliations:** ^1^ Institute for Biodiversity and Ecosystem Dynamics (IBED) University of Amsterdam P.O. Box 94248, 1090 GB Amsterdam The Netherlands; ^2^ Department of Evolutionary Biology and Environmental Studies University of Zurich Winterthurerstrasse 190, 8057 Zurich Switzerland; ^3^ Department of Fish Ecology & Evolution, Eawag Swiss Federal Institute for Aquatic Science and Technology 6047 Kastanienbaum Switzerland; ^4^ German Centre for Integrative Biodiversity Research (iDiv) Halle‐Jena‐Leipzig Deutscher Platz 5e Leipzig 04103 Germany

**Keywords:** Adaptation, direct development, evolution, life‐history evolution, metamorphosis, size structure

## Abstract

Although metamorphosis is widespread in the animal kingdom, several species have evolved life‐cycle modifications to avoid complete metamorphosis. Some species, for example, many salamanders and newts, have deleted the adult stage via a process called paedomorphosis. Others, for example, some frog species and marine invertebrates, no longer have a distinct larval stage and reach maturation via direct development. Here we study which ecological conditions can lead to the loss of metamorphosis via the evolution of direct development. To do so, we use size‐structured consumer‐resource models in conjunction with the adaptive‐dynamics approach. In case the larval habitat deteriorates, individuals will produce larger offspring and in concert accelerate metamorphosis. Although this leads to the evolutionary transition from metamorphosis to direct development when the adult habitat is highly favorable, the population will go extinct in case the adult habitat does not provide sufficient food to escape metamorphosis. With a phylogenetic approach we furthermore show that among amphibians the transition of metamorphosis to direct development is indeed, in line with model predictions, conditional on and preceded by the evolution of larger egg sizes.

What do the Puerto Rican tree frog, *Eleutherodactylus coqui*; the sea urchin, *Abatus cordatus*; and the flat periwinkle (*Littorina obtusata*, a marine sea snail) have in common? Their offspring are all born with the adult morphology and do not metamorphose. Somewhere in their evolutionary history, the ancestors of these species evolved direct development and lost the ability to metamorphose. Why did this life‐history strategy evolve?

Even though some species, including humans, have direct development, metamorphosis is the dominant life‐history strategy in the animal kingdom (Werner 1988). We define metamorphosis here as the morphological change that takes place at the transition from the free‐living larval to the juvenile stage. This morphological change allows for the effective exploitation of different niches during an individual's life (Moran 1994). As metamorphosing species often depend on multiple niches for their growth and reproduction, they are vulnerable to habitat degradation because a metamorphosing population can already go extinct if only one of the two habitats becomes unsuitable (Rudolf and Lafferty 2011). Metamorphosis is furthermore both a risky and energetically costly process (e.g., Wassersug and Sperry 1977; Geffen et al. 2007). It is therefore likely that under some ecological conditions individuals evolve a life‐history strategy without metamorphosis.

Metamorphosis can be lost via the evolution of either paedomorphosis or direct development. Paedomorphosis, where individuals retain the larval features during their whole life cycle, is common in salamanders (Denöel et al. 2005) but, for example, absent in frogs (Elinson and del Pino 2012). In direct developing species, the adult features form during the embryonic stage and are present at hatching (Callery et al. 2001). Species with direct development lack a free‐living larval stage. Direct development evolved at least 10 times in anurans (Hanken 1999) and at least twice in salamanders (in the lungless salamanders, Wake and Hanken 1996). Direct development is also a common life‐history strategy among marine invertebrates (e.g., Marshall et al. 2012) and the default strategy among mammals.

Although there are many studies that describe the morphological and hormonal development of direct developing species (e.g., Callery et al. 2001; Schweiger et al. 2017; Helm 2018), from an ecological point of view it is not well understood how and why direct development evolved. It is likely that unfavorable conditions for larvae select for the evolution of direct development. Life‐history data of marine invertebrates, for example, show that aplanktonic species, where individuals are born with the adult morphology, are more common in unproductive larval environments (Fernández et al. 2009; Marshall et al. 2012). Empirical data furthermore show that direct development is associated with the production of larger offspring (e.g., Raff 1987; McEdward 2000; Callery et al. 2001; Marshall et al. 2012), but it is unknown if direct development leads to the evolution of larger offspring or the other way around.

In a previous study (ten Brink et al. 2019), we focused on the ecological conditions promoting the evolution of metamorphosis. In addition, we showed that after metamorphosis has evolved, a population does not often abandon this life‐history strategy when ecological conditions change, not even when this leads to the extinction of the population. In that study, we investigated the evolutionary response of a consumer population to changes in the productivity of the adult habitat. As we assumed that newborn individuals needed to feed on the primary food source to grow, it was in this study impossible to lose the larval stage.

The focus of the current article is to understand the ecological conditions favoring the evolution of direct development in an initially metamorphic population. We study how such a metamorphosing population responds to a deteriorating larval habitat, using similar models as in ten Brink et al. (2019). To allow individuals to lose the larval stage, we assume that the body mass at birth can evolve. In these size‐structured consumer‐resource models, consumers forage on two types of food. These food sources require different morphologies to be effectively used. Although large individuals can feed upon both food sources, small individuals can feed only upon the primary food source because they are too small to handle the secondary food source. Individuals are born with a morphology specialized in feeding on the primary food source. At a certain body mass individuals undergo metamorphosis and develop a morphology specialized in feeding on the secondary food source. Although metamorphosis allows for the efficient exploitation of the two food sources, we assume that it is an energetically costly process. We study the evolutionary response of a metamorphosing population in relation to changes in the supply rate of the primary food source. As the benefits and costs of metamorphosis depend on the densities of the two food sources and these densities are in turn affected by the strategy of the consumers, it is important to take into account the feedback loop between the environment and the consumer individuals. We therefore use the framework of adaptive dynamics (Geritz et al. 1998) to study the evolutionary loss of metamorphosis via direct development.

We first study how metamorphosing individuals will respond to changes in the supply rate of the primary food source. We find that there is selection to produce larger offspring when the primary food source becomes less productive. Second, we study under which ecological conditions metamorphosis can disappear through the evolution of direct development. We find, as before (ten Brink et al. 2019), that metamorphosis is hard to lose, even when this leads to the extinction of the population. We show that only when the adult habitat is highly productive, direct development can evolve. Finally, we test our predictions regarding the evolution of direct development in amphibians with the use of a phylogenetic comparative framework (Pagel 1994). We show that the evolutionary transition from metamorphosis to direct development was dependent on the evolution of large eggs, consistent with the results from our theoretical model.

## Model and Methods

### MODEL DESCRIPTION

To understand under which ecological conditions metamorphosis can disappear via the evolution of direct development, we use two size‐structured consumer‐resource models. In both models, growth and fecundity of an individual depend on the body size of the individual and on its food intake. Metamorphosis is typically an energetically costly process, where individuals lose part of their fat reserves (Geffen et al. 2007). To capture this process, a model is needed that takes into account that individuals consists of irreversible mass, such as bones and organs, and reversible mass, such as fat and gonads. We therefore use an adapted version of the size‐structured consumer‐resource model described by Persson et al. (1998), which we refer to as the fat‐reserves model. This model is based on the interaction between roach (*Rutilus rutilus*) and two small‐bodied zooplankton species. The model depends on a large number of parameters and has some specific functions (e.g., hump‐shaped attack‐rate functions). Although the model therefore has a clear basis in biological reality, not all assumptions may apply to other systems.

To assess the generality of our results, we therefore also study the evolution of direct development in a more abstract, generic size‐structured model. In this model, individuals are fully characterized by their size, and we do not distinguish between different types of body mass. Metamorphosis is hence modeled in a phenomenological way, where individuals do not lose body fat, but become smaller after metamorphosis. In addition to modifying the model structure, we parameterize this model for invertebrate species, following de Roos and Persson (2013).

**Figure 1:**
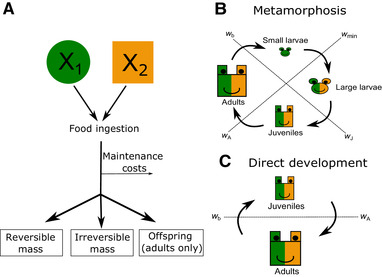
Schematic diagram of the energy flows (panel A) and life cycles (panels B and C) in the model. (A) Ingested food is first used to cover maintenance costs. Subsequently, the net‐biomass production is allocated to reversible and irreversible body mass. Adults also allocate energy to reproduction. In panels B and C, the colors of the individuals in the life cycles indicate to which food sources they have access to (dark green for primary food source, orange for secondary food source). The shape of the individuals indicate on which food source they are specialized (circles are specialized on primary food source, squares are specialized on secondary food source). (B) Life‐history diagram of individuals with metamorphosis. Newborn individuals have access to the primary food source only. After reaching a body mass of $w_{\min}$ they can feed on the secondary food source as well. However, larvae have a morphology specialized in feeding on the primary food source and are therefore not efficient in feeding upon the secondary food source. After metamorphosis (at a body mass of $w_{J}$), individuals have a morphology specialized in feeding upon the secondary food source. Individuals mature when reaching a body mass of $w_{A}$ (C) Life‐history diagram of individuals with direct development. Newborn individuals are born with a morphology specialized in feeding upon the secondary food source. Individuals do no longer undergo metamorphosis.

Because of its biological realism, in the main text we focus on the fat‐reserves model. Next, we describe the most important aspects of this model, a detailed model description can be found in ten Brink et al. (2019) and Methods S1. A schematic overview of the model for individual consumers can be found in Figure 1. A detailed model description of the generic size‐structured model can be found in Methods S2. We study this model in “Generality of Results” in the Appendix. In Table S3.1, we give an overview of the differences and similarities of the fat‐reserves model and the generic size‐structured model.

We assume that consumers have access to two food sources that each require a different morphology to be effectively used. The primary food source is available for all consumers, whereas the secondary food source is available only for individuals with a body mass larger than $w_{\min}$. Both primary and secondary food sources follow semichemostat dynamics with a turnover rate of δ and will, in the absence of consumers, reach a maximum density of $X_{1,\max}$ and $X_{2,\max}$, respectively.

Initially, the consumer has three life stages; larvae (L), juveniles (J), and adults (A). Individuals are characterized by two different forms of mass, irreversible mass x such as bones and organs and reversible mass y such as fat. The total body mass, w, of an individual is determined by the sum of the reversible and irreversible mass, w=x+y. Larvae are born with body mass $w_{b}$ and metamorphose into juveniles at a body mass of $w_{J}$, juveniles subsequently mature into adults and start reproducing when reaching a body mass of $w_{A}$ (panel B in Fig. 1). The morphology of an individual is characterized by the relative degree of specialization $\psi_{i}$ (i= L, J, or A) on the secondary food source; a value of $\psi_{i}=0$ means that individuals are completely specialized in feeding on the primary food source, conversely, a value of $\psi_{i}=1$ means that individuals are completely specialized in feeding on the secondary food source.

Food ingestion is size‐dependent and furthermore depends on food densities following a saturating functional response (ten Brink et al. 2019). The attack rates are hump‐shaped functions of the body size of the consumer. The maximum height of these hump‐shaped functions is determined by the relative degree of specialization $\psi_{i}$ (i= L, J, or A) on the secondary food source. Handling times are equal for both food sources and depend only on the body mass of the consumer. The preference of individual consumers to feed on either of the two food sources depends on their encounter rates following optimal foraging considerations. Ingested food is assimilated with a constant efficiency $\kappa_{e}$ and subsequently used for covering basic maintenance costs, growth, and, in the case of adults, reproduction (panel A in Fig. 1). There is a trade‐off between the number of offspring an individual produces and the body mass of its newborn larvae. The larger the body mass of the offspring, the lower the fecundity rate of the mother.

Metamorphosis decouples the morphologies expressed at different life stages such that an individual can adopt different morphologies before and after metamorphosis. The morphology of larvae is fully determined by parameter $\psi_{L}$. The morphology of postmetamorphs (juveniles and adults) is determined by two traits, the extent of metamorphosis θ and the larval specialization parameter $\psi_{L}$ following

(1) $$\psi_{A}=\psi_{J}=\min\left(1,\psi_{L}+\theta \right).$$

Note that $\psi_{L}$, $\psi_{J}$, $\psi_{A}$, and θ have values between 0 and 1. Individuals that undergo metamorphosis lose part of their body mass and furthermore have a probability of ρθ to die during metamorphosis. We refer to the larval morphology in case a life stage is specialized in feeding on the primary food source ($\psi_{i}=0$) and to the adult morphology in case a life stage is (partly) specialized in feeding on the secondary food source ($\psi_{i}>0$).

In case the body mass at birth $w_{b}$ evolves to values larger than the body mass at metamorphosis, metamorphosis takes place before individuals are born. In this case the mother pays for the cost of the metamorphosis of her offspring, such that her fecundity linearly declines with the degree of metamorphosis θ (see Methods S1).

To understand how direct development can evolve from metamorphosis we study the evolution of four traits (Table 1); the two traits that determine the morphology of an individual over its lifetime ($\psi_{L}$ and θ), the body mass at metamorphosis $w_{J}$ and the body mass at birth $w_{b}$. We use the framework of adaptive dynamics to study the evolution of these four traits (Geritz et al. 1998). Adaptive dynamics assumes that mutations have only small phenotypic effects. These small mutations occur infrequently, such that the previous mutant has either been established or disappeared and that the ecological environment has reached an attractor by the time a new mutant appears. The success of a mutant depends on its strategy and on the environment it encounters. In “Results of the Individual‐Based Model”in the Appendix, we show with an individual‐based model how relaxing these assumptions of adaptive dynamics affects our results.

**Table 1:** Evolving traits in the fat‐reserves model

Variable	Description	Range	Unit
$\psi_{L}$	Degree of specialization of larvae on the secondary food source	From 0 to 1	–
θ	Extent of metamorphosis	From 0 to 1	–
$w_{J}$	Standardized body mass at metamorphosis	Larger than 0.0001	g
$w_{b}$	Standardized body mass of newborns	Larger than 0.0001	g

We assume that initially larvae are completely specialized on the primary food source ($\psi_{L}=0$) whereas postmetamorphs are (partly) specialized on the secondary food source (θ>0), which is the case when the supply rates of both food sources are high (ten Brink et al. 2019). To understand which ecological conditions lead to the disappearance of metamorphosis, we track this evolutionary singular strategy (ESS) predicted by the model for decreasing values of the supply rate of the primary food source, $\delta X_{1,\max}$. We decrease the supply rates by varying $X_{1,\max}$ while keeping δ constant. By tracking the ESS, we assume that the change in the supply rate is relatively slow and that evolution is able to track this change in ecological conditions. We relax this assumption in “Results of the Individual‐Based Model” in the Appendix. We assume that the body mass at which the secondary food source becomes available $w_{\min}$ does not evolve. As this parameter is possibly of importance for the evolutionary outcome, we also investigate the effect of different values for this parameter.

For most values of $w_{\min}$, the ESSs found possess strong convergence stability and therefore correspond to a continuously stable strategy (CSS) (Leimar 2009). This implies that the traits will evolve to the singular strategy, and after they reach this point, the traits will not change over evolutionary time. However, in case $w_{\min}$ is low and the supply rates of both food sources are high, the ESSs are no longer convergence stable. Instead, the four evolving traits always change over evolutionary time and fluctuate around a fixed value. The reason that the traits always keep evolving is that the selection gradient vanishes for an ecological steady state that is dynamically unstable (saddle point) and hence not an ecological attractor. In “Evolutionary Cycling” in the Appendix, we show with the canonical equation of adaptive dynamics (Dieckmann and Law 1996; Durinx et al. 2008) how the four traits evolve in this case. For simplicity we use the identity matrix for the mutational covariance matrix. This implies that an evolutionary change in one trait, will not directly affect another trait, all traits therefore evolve independently from each other. Even though the four evolving traits always vary over evolutionary time, they stay close to the strategy with a vanishing selection gradient that gives rise to an ecologically unstable steady state. We therefore ignore this subtlety in the result section and will refer to the strategy with a vanishing selection gradient as an ESS irrespective of its ecological instability.

All analyses were performed using the PSPManalysis software package (de Roos 2016). This software package allows for the equilibrium and evolutionary analysis of physiologically structured population models (see Kirkilionis et al. 2001; Diekmann et al. 2003 and de Roos 2008 for more details). The model‐specific files needed for PSPManalyis together with an R script that executes all the calculations made in this article are available in the Dryad data repository.

### PHYLOGENETIC COMPARATIVE ANALYSES

We used a phylogenetic comparative analysis to test the correlation between the evolution of direct development and offspring size. A dated phylogenetic tree including 2871 amphibian species was obtained from Pyron and Wiens (2013). We obtained data for direct development and egg size, as a proxy for offspring size, from the AmphiBIO database (Oliveira et al. 2017) and matched this against the phylogenetic data. From the entries in this database we used breeding strategy “Dir” to indicate whether species reproduce via direct development or not (binary). We used “Offspring_size_min_mm” as a measure of egg size. As egg size is a continuous trait, and our analyses (see below) can handle only binary data, we defined large eggs as greater than or equal to the average across all amphibians in the AmphiBIO database for which egg size data were available, and small eggs as less than the average. The average was 2.45 mm. The AmphiBIO database does not provide information on the developmental mode of species with a viviparous (live‐bearing) breeding strategy (Viv). We therefore collected this information from amphibiaweb.org. Even though the AmphiBIO database includes data on offspring size of these viviparous species, we excluded these data in our analysis because offspring size is not comparable to egg size. In total, we obtained data on developmental mode for 79% of the species (n = 2261) and egg size data for 28% of the species (n = 795) for which we also had phylogenetic data (n = 2871).

We tested for correlated evolution between direct development and large egg sizes in a phylogenetic comparative framework (Pagel 1994). To do so, we calculated the log marginal likelihood of an independent and a dependent model. In the independent model we assumed that transitions in the two traits (between no direct development and direct development and between small eggs and large eggs) occurred completely independently from each other. In this independent model, the evolution of direct development does not depend on the presence of large eggs and, vice versa, the evolution of large eggs is independent of the type of developmental mode. We compared the fit of this model with a dependent model where the evolution of the two traits was correlated. In this model, the transition rates of both traits depend on the state of the other trait. Here we assumed that the probability that two traits change at exactly the same time equals zero (Pagel 1994). There are therefore in total eight transition rates calculated. Note that we also calculated the transition rates at which a population with direct development will reevolve metamorphosis, even though with our size‐structured population models we do not make any predictions regarding this evolutionary transition. All phylogenetic analyses were carried out in BAYESTRAITS v3 (Meade and Pagel 2017).

We ran five replicate Markov chain Monte Carlo (MCMC) chains for models of independent evolution and dependent (correlated) evolution, using a reversible jump hyper prior with an exponential prior between 0 and 100 and using a stepping stone sampler (Xie et al. 2011) to obtain estimates of the log marginal likelihoods. These MCMC chains were run for 5,000,000 generations and we discarded a 10% burn‐in. Support for correlated evolution was calculated using log Bayes factors as follows:

(2) $$\begin{array}{ccc} & & 2\cdot \mathrm{(log}\;\;\text{marginal}\;\;\text{likelihood}\;\;\text{(dependent}\;\;\mathrm{model)}\\ & & \;-\log\;\;\text{marginal}\;\;\text{likelihood}\;\text{(independent}\;\;\mathrm{model)).} \end{array}$$

A log Bayes factor >2 indicates support and scores >10 indicate very strong support for the dependent model and thus for correlated evolution (Kass and Raftery 1995).

We evaluated transition rates to assess whether the transition toward direct development is conditional on the evolution of large egg sizes. The significance of this was tested by comparing Bayes factors of the full, dependent model (no constraints) to a constrained model. In this constrained model, we assumed that large eggs and small eggs may equally likely be present when direct development evolves. The constrained model therefore only calculates seven transition rates (in contrast to eight transition rates in the full, dependent model). We compared again the log marginal likelihoods of both models to test which model fits the data the best (Pagel 1994).

## Results

In the first part of this section, we show that individuals produce larger offspring when the primary food source deteriorates. Although this sometimes leads to the evolutionary transition of metamorphosis to direct development, the population often goes extinct when the primary food source becomes too scarce. In the second section, we show how the evolution of direct development depends on the supply rate of the secondary food source and on parameter $w_{\min}$, which determines at which body mass this food source becomes available. In the last section, we demonstrate with a phylogenetic comparative analysis that the evolution of large egg sizes preceded the evolution of direct development in amphibians.

### EVOLUTION OF LARGER OFFSPRING WHEN THE PRIMARY FOOD SOURCE DETERIORATES

**Figure 2:**
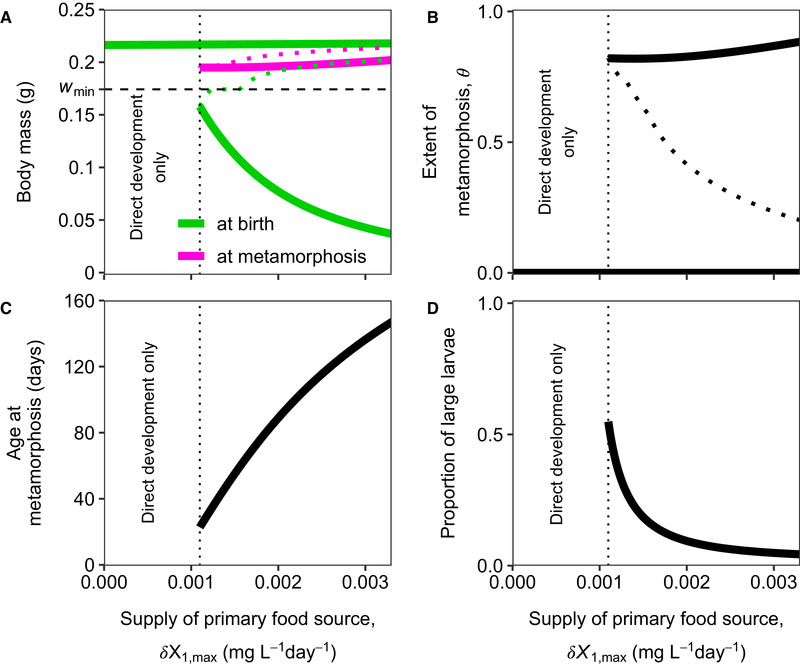
For high supply rates of the secondary food source, a metamorphosing population evolves direct development for low values of the supply rate of the primary food source. (A) Body mass (g) at birth ($w_{b}$, green) and body mass at metamorphosis ($w_{J}$, light purple), (B) the extent of metamorphosis (θ), (C) age at metamorphosis (days), and (D) proportion of large larvae at the ESSs as a function of the supply rate of the primary food source ($\mathrm{mg}\;L^{-1}{\mathrm{day}}^{-1}$). Solid lines indicate CSSs, whereas the dotted lines in (A) and (B) indicate evolutionary repellers. The horizontal green line in (A) shows the body mass at birth at the ESS for a population with direct development, which hence does not depend on the primary food source. As this population does not undergo metamorphosis (θ=0, $\psi_{L}=1$), body mass at metamorphosis is undefined and therefore not plotted. For high supply rates of the primary food source (around 0.0087 $\mathrm{mg}\;L^{-1}{\mathrm{day}}^{-1}$), the life‐history strategy with direct development becomes evolutionary unstable and is no longer an ESS (not shown). The proportion of large larvae is calculated as the numerical abundance of larvae that have access to the secondary food source (with body mass $w_{\min}<w<w_{J}$) divided by the numerical abundance of all larvae (with body mass $w<w_{J}$). The vertical dotted lines in all panels indicate at which value of the supply rate the population evolves direct development. The black dashed line in panel (A) indicates the body mass at which the secondary food source is available ($w_{\min}$). Population and food densities as a function of the supply rate of the primary food source are plotted in Figure S5.1. The supply rate of the secondary food source equals $\delta X_{2},\max=0.0165\;\mathrm{mg}\;L^{-1}{\mathrm{day}}^{-1}$. Other parameter values are as shown in Tables S1.2 and S1.3.

When the primary food source deteriorates, there is selection to reduce the period where individuals depend on this food source. There is therefore an evolutionary response to a diminishing supply rate of the primary food source such that individuals produce larger offspring and furthermore metamorphose at a smaller body mass (panel A in Figs. 2 and 4). By increasing the body mass at birth $w_{b}$ and decreasing the body mass at which individuals undergo metamorphosis $w_{J}$, individuals will metamorphose at an earlier age (panel C in Figs. 2 and 4). This evolutionary response will furthermore lead to a higher proportion of larvae that no longer depend on the primary food source for their survival and growth (panel D in Figs. 2 and 4).

**Figure 3:**
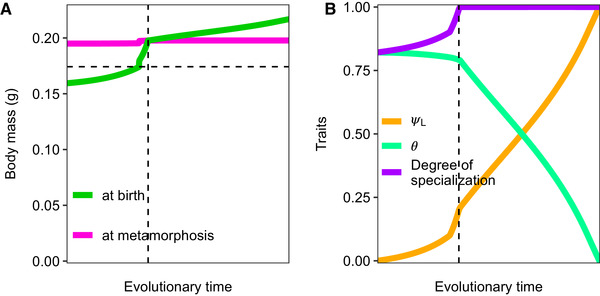
For high supply rates of the secondary food source, a metamorphosing population evolves direct development for low values of the supply rate of the primary food source. Evolutionary dynamics, starting from a metamorphic initial population, of (A) the body mass at birth ($w_{b}$, green) and at metamorphosis ($w_{J}$, light purple) in grams, and of (B) the extent of metamorphosis (θ, mint green), the larval specialization parameter $\psi_{L}$ (orange), and the resulting specialization on the secondary food source for individuals with body mass $w\geq w_{J}$ (dark purple). The vertical dashed line indicates the moment where direct development evolves via internalization (panel C in Fig. 1). The supply rate of the secondary food source equals $\delta X_{2},\max=0.0165\;\mathrm{mg}\;L^{-1}{\mathrm{day}}^{-1}$, that of the primary food source $\delta X_{1,\max}=0.0011\;\mathrm{mg}\;L^{-1}{\mathrm{day}}^{-1}$, which is the density at which direct development can evolve (vertical dotted line in Fig. 2). The horizontal dashed line in panel (A) indicates the body mass at which the secondary food source is available ($w_{\min}$). Other parameter values are as shown in Tables S1.2 and S1.3.

**Figure 4:**
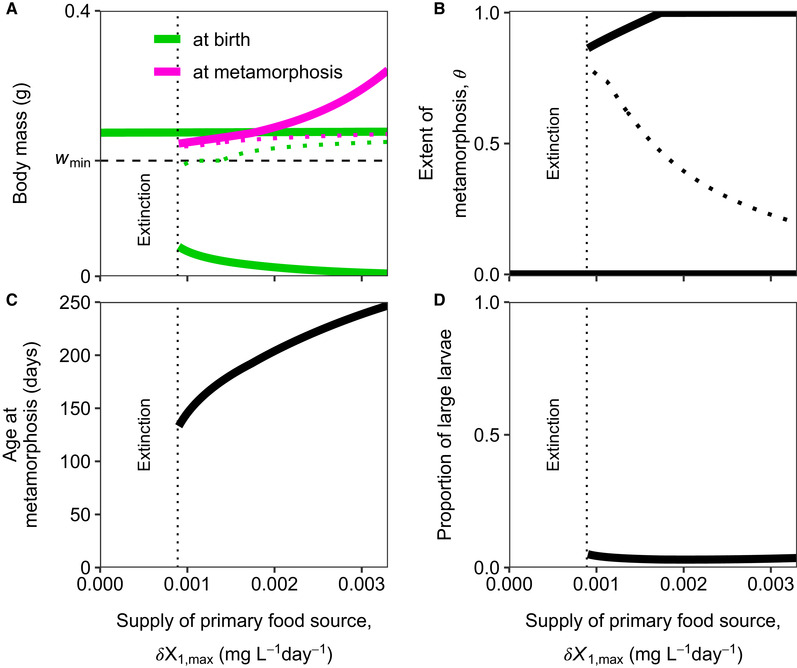
For low supply rates of the secondary food source, a metamorphosing population goes extinct for low values of the supply rate of the primary food source. (A) Body mass (g) at birth ($w_{b}$, green) and body mass at metamorphosis ($w_{J}$, light purple), (B) the extent of metamorphosis (θ), (C) age at metamorphosis, and (D) proportion of large larvae at the ESSs as function of the supply rate of the primary food source ($\mathrm{mg}\;L^{-1}{\mathrm{day}}^{-1}$). Solid lines indicate continuously stable strategies whereas the dotted lines in (A) and (B) indicate evolutionary repellers. The horizontal green line in (A) shows the mass at birth at the ESS for a population with direct development, which hence does not depend on the primary food source. As this population does not undergo metamorphosis (θ=0, $\psi_{L}=1$), body mass at metamorphosis is obsolete and therefore not plotted. For high supply rates of the primary food source (around 0.009 $\mathrm{mg}\;L^{-1}{\mathrm{day}}^{-1}$), the life‐history strategy with direct development becomes evolutionary unstable and is no longer an ESS (not shown). The proportion of large larvae is calculated as the numerical abundance of larvae that have access to the secondary food source (with body mass $w_{\min}<w<w_{J}$) divided by the numerical abundance of all larvae (with body mass $w<w_{J}$). The vertical dotted lines in all panels indicate at which value of the supply rate the population goes extinct. The black dashed line in panel (A) indicates the body mass at which the secondary food source is available ($w_{\min}$). Population and food densities as a function of the supply rate of the primary food source are plotted in Figure S5.2. The supply rate of the secondary food source equals $\delta X_{2},\max=0.0066\;\mathrm{mg}\;L^{-1}{\mathrm{day}}^{-1}$. Other parameter values are as shown in Tables S1.2 and S1.3.

As individuals depend crucially on the primary food source when their body mass is smaller than $w_{\min}$, it is of importance to produce offspring that are large enough to immediately start feeding on the secondary food source when the primary food source is very scarce or even absent. Producing large offspring is energetically more expensive than producing small offspring. Adults that produce larger offspring therefore need more food to produce a single offspring compared to adults that produce smaller offspring. Hence, there is a trade‐off between producing many small individuals that depend on the primary food source for a long time or producing a few big individuals that do not rely on this food at all. We will consider two scenarios: when the supply rate of the secondary food source is high (Figs. 2 and 3) and when it is low (Fig. 4).

When the supply rate of the secondary food source is high, direct development can evolve from metamorphosis (Figs. 2 and 3). Figure 2 shows how the evolutionary equilibrium changes as a function of the supply rate of the primary food source. As the supply rate of the primary food source diminishes, the body mass at birth increases (Fig. 2A), such that for low supply rates a large proportion of the larvae no longer depend on the primary food source (Fig. 2D). Because many larvae can now also feed upon the abundant secondary food source, there is for low supply rates of the primary food source (vertical dotted line in Fig. 2) selection for larvae to specialize upon the secondary food source and therefore to increase specialization parameter $\psi_{L}$. The CSS where metamorphosis is present merges with the evolutionary repeller (dotted lines in Figs. 2A and B) and metamorphosis disappears (Fig. 2B). The population now evolves to the alternative direct developing life‐history strategy where there is no longer metamorphosis (horizontal green line in panel A and θ=0 in panel B of Fig. 2).

Figure 3 shows how the evolving traits change over evolutionary time as soon as the CSS in which metamorphosis is present disappears. Because many larvae are at this point able to feed on the secondary food source (Fig. 2C), there is selection to increase the specialization parameter $\psi_{L}$ (orange line in Fig. 3B). Simultaneously, the body mass at birth increases (Fig. 3A), such that at a certain point in time the body mass at birth is larger than the body mass at which the secondary food source is available ($w_{\min}$, vertical dashed line in Fig. 3A). At this point, individuals no longer rely on the primary food source. The body mass at birth increases further and direct development evolves the moment metamorphosis takes place before individuals are born (vertical dotted line in Fig. 3). Because metamorphosis is still costly (subsumed into the costs that the mother makes to produce a single offspring, see Methods S1), there is selection to reduce the extent of metamorphosis θ (mint‐green line) while at the same time it is beneficial to increase specialization parameter $\psi_{L}$ (orange line) such that all individuals have a morphology fully specialized on the secondary food source (dark purple line in Fig. 3B). As individuals no longer pay the costs of metamorphosis, the body mass at birth will evolve to slightly higher values (Fig. 3A) because adults have more energy available to produce large offspring.

For low supply rates of the secondary food source, there is again an evolutionary response to produce larger offspring and metamorphose at a smaller body mass with decreasing supply rates of the primary food source (Fig. 4A). However, because of the low supply rate of the secondary food source, the density of this food is not high enough for metamorphosing individuals to produce larvae large enough to completely skip the primary food source (Fig. 4A). As larvae depend on the primary food source most of their life (Fig. 4D), there is no selection to specialize on the abundant secondary food source. The population therefore goes extinct in case of diminishing supply rates of the primary food source. Nonetheless, for these low supply rates of the secondary food source there is an alternative, viable, evolutionary attractor, where individuals have direct development (θ=0 and $\psi_{L}=1$; Fig. 4B) and hence do not depend on the primary food source. The individuals in this case have a CSS value for body size at birth just above $w_{\min}$ (horizontal line in Fig. 4A). However, a metamorphosing population never evolves toward this strategy because the evolutionary attractor of the metamorphosing population collides with its extinction boundary for low supply rates of the primary food source.

### WHEN DOES DIRECT DEVELOPMENT EVOLVE?

In the previous section, we showed the evolutionary response of a metamorphosing population to diminishing supply rates of the primary food source. In this section, we show how these results depend on the supply rate of the secondary food source and the body mass at which this food source becomes available.

**Figure 5:**
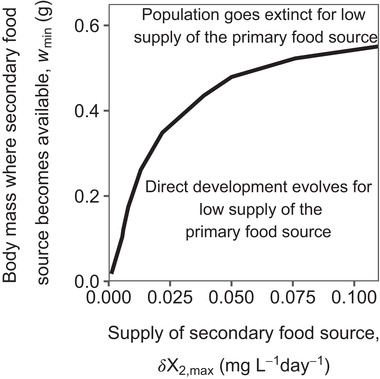
The smaller the body mass where the secondary food source is available ($w_{\min}$), the easier it is to evolve direct development. Two‐parameter plot showing where direct development can evolve. The horizontal axis shows the supply rate of the secondary food source ($\delta X_{2},\max(\;\mathrm{mg}\;l^{-1}{\mathrm{day}}^{-1}$)), the vertical axis shows the body mass at which this food source becomes available ($w_{\min}$ (gram)). Below the black line, direct development can evolve for low supply rates of the primary food source. A metamorphosing population goes extinct when the supply rate of the primary food source decreases for parameter combinations above the black line. Parameter values are as shown in Tables S1.2 and S1.3.

When the secondary food source is already available early in life, direct development almost always evolves from metamorphosis when the supply rate of the primary food source diminishes (Fig. 5). Direct development can evolve easily because individuals can skip the primary food source even when they are born with a relatively small body mass. For very low supply rates of the secondary food source, however, adults do not have enough food available to produce offspring large enough to skip the primary food and the population goes extinct in case the supply rate of this food becomes too low. When the secondary food source is available late in life, direct development can evolve only when the supply rate of the primary food source is very high, otherwise the population goes extinct for low supply rates of the primary food source. Adults can produce offspring large enough to skip the primary food source only when there is a lot of the secondary food source available.

To summarize, a metamorphosing population easily goes extinct in case the primary food source diminishes. Direct development can evolve from metamorphosis in case the secondary food source is available early in life and when the supply rate of this food source is high. The earlier the secondary food source is available, the lesser the supply rate of this food source necessary for direct development to evolve.

### GENERALITY OF RESULTS

In “Generality of Results” in the Appendix, we analyze the evolutionary transition of metamorphosis to direct development in the generic size‐structured model parameterized for invertebrate species. The analysis of this model shows that our results are robust against major changes in model structure and parameters. The results of the generic model differ only in one minor aspect from the results of the fat‐reserves model. In the fat‐reserves model, we find that there is an alternative, viable, life‐history strategy of direct development present for conditions under which a metamorphosing population goes extinct. This is not the case in the generic size‐structured model. The presence of this evolutionary bistability is, however, not so relevant because in the fat‐reserves model, a metamorphosing population will not evolve to this alternative evolutionary equilibrium. It is, therefore, beyond the focus of this article to study why the models differ in this aspect.

### DIRECT DEVELOPMENT ONLY EVOLVES IN AMPHIBIANS AFTER THE EVOLUTION OF LARGE EGGS

Our model results show that before direct development evolves, there is selection for increased offspring size (Fig. 2A). Although a correlation between developmental mode and offspring size has been observed before (e.g., McEdward 2000; Callery et al. 2001; Marshall et al. 2012), this correlation has not been tested in a phylogenetic framework.

We found very strong support for correlated evolution between direct development and large egg sizes within the amphibians, that is, the average log Bayes factor over all five parallel runs was 21.8 in favor of the dependent model (Table A1). To test if the transition from metamorphosis to direct development indeed depends on the presence of large eggs, we furthermore compared the Bayes factor of the fully dependent model to a constrained model where we assumed that the transition rate to direct development does not depend on egg size. The dependent model performed better than the model in which we constrained the transition to direct development to be independent of egg size (Bayes factor of 2.99).

Table 2 shows the conditional transition rates of the two traits, estimated by the dependent model. The presence of direct development is indicated with D = 1, its absence with D = 0. Large eggs are referred to as E = 1, small eggs as E = 0. The parameter P(E = 1 → 0 | D = 1), for example, is the estimated transition probability per million years from large to small eggs in case individuals have direct development. These transition rates strongly support the prediction that direct development cannot evolve unless large egg size has evolved first, that is, the transition from metamorphosis to direct development is zero if small eggs are present. The transition rates furthermore show that the loss of direct development is impossible when the lineage has large egg sizes. All other transition scenarios are equally likely (Table 2). It is therefore for example possible that after direct development has evolved, small eggs evolve again.

**Table 2:** Transition probabilities per million years between developmental modes (trait *D*) and large egg size (trait *E*) for the correlated (dependent) model of evolution resulting from Bayesian Markov chain Monte Carlo analyses in BayesTraits. The entries in bold show the transition rates to direct development

Transition rate	Median Estimate (Transitions/Million Years)
**P(D = 0** → **1** | **E = 0)**	0
**P(D = 0** → **1** | **E = 1)**	0.004425
*P*(*D* = 1 →0 | *E* = 0)	0.0044915
*P*(*D* = 1 →0 | *E* = 1)	0
*P*(*E* = 0 →1 | *D* = 0)	0.00499
*P*(*E* = 0 →1 | *D* = 1)	0.0049175
*P*(*E* = 1 →0 | *D* = 0)	0.005004
*P*(*E* = 1 →0 | *D* = 1)	0.004683

*Note*: “0” refers to absence of the trait, “1” refers to presence.

## Discussion

In this article we showed that a metamorphosing population evolves in response to changing conditions in such a way that individuals reduce their dependence on the food source on which small individuals rely. Although this can lead to the evolution of direct development, we also found that metamorphosis tends to be an evolutionary dead end. These results are comparable to the results of a previous study (ten Brink et al. 2019), where we studied the response of a metamorphosing population to deteriorating conditions in the adult habitat. There, we also found that a metamorphosing population will often go extinct in case the food source on which adult individuals rely becomes insufficient to sustain the population. As metamorphosing species often crucially depend on two (or more) habitats for their growth, survival, and reproduction, they are sensitive to habitat loss (Rudolf and Lafferty 2011) and a metamorphosing population often goes extinct when one of the food sources on which they rely becomes too scarce.

Our results demonstrate that under limited ecological conditions direct development can evolve, which shields species from extinction in case of severe habitat degradation. However, in our analysis we assumed an infinite population size and ignored stochastic processes. It is therefore likely that metamorphosing populations are more vulnerable to habitat degradation than our deterministic model suggests. In “Results of the Individual‐Based Model” in the Appendix, we show with the use of an individual‐based model that this is indeed the case. We show that a metamorphosing population sometimes goes extinct even when the supply rate of the secondary food source is high enough for direct development to evolve. The results from the individual‐based model suggest that in case ecological conditions allow for the evolution of direct development, this evolutionary transition is more likely to happen when the mutation rate is high and habitat degradation slow, such that the population can adaptively track the change in environmental conditions. We furthermore show in “Results of the Individual‐Based Model” in the Appendix that large populations are more likely to evolve direct development compared to small populations (Claessen et al. 2007, 2008).

The results in our study strongly depend on the feedback between individual development, ecology, and evolution. When the supply rate of the primary food source decreases, competition for this food source becomes intense for small larvae, which depend on this food for their growth. Fecundity of adults depends on food intake as well. When the secondary food source is in high supply, competition for this food is limited, allowing adults to produce many large offspring. Even though these large offspring initially compete for the scarce primary food source, they do not have to grow much before they are big enough to access the secondary food source. As they get access to this abundant secondary food source early in life, there is selection to specialize on this food source and a life‐history strategy with direct development will evolve. However, when the supply rate of the secondary food source is low, competition among adults is strong as well. They therefore produce small offspring that have to grow a lot before they are big enough to access the secondary food source. When there is little of the primary food source available, growth is slow and it takes therefore a long time before they reach this body mass. In addition, the benefit of specializing on the secondary food source is small because competition for this food source is strong as well. A mutant larvae that would be less specialized on this primary food source, will be outcompeted by the residents (ten Brink and de Roos 2017). Therefore, there will be strong selection to be highly specialized on the scarce primary food source and direct development cannot evolve. Hence, the evolutionary transition from metamorphosis to direct development strongly depends on the coupling between individual development, evolutionary dynamics, and ecological dynamics, which should therefore not be ignored.

In case of low supply rates of the secondary food source, a metamorphosing population goes extinct for low supply rates of the primary food source. This evolutionary trap occurs for parameter values for which also a viable evolutionary attractor exists, characterized by the absence of metamorphosis. Even though it could be possible that a metamorphosing population escapes the evolutionary trap by evolving to this alternative strategy, we never encountered such a result in our individual based simulations, not even when large mutational steps are allowed. The reason for this is that for most values of the supply rate of the primary food source, the metamorphosing population suppresses the densities of the secondary food source to such low levels that a direct developer cannot survive (see Fig. S5.2). Therefore, even if a direct developer evolves due to some big mutational step, it will not be able to establish itself in a population with metamorphosing individuals. Only close to the extinction boundary of the metamorphosing population the density of the secondary food source is high enough for a mutant with direct development to successfully invade a metamorphosing population. However, for these low supply rates of the primary food source, a metamorphosing population is small, which increases the chance that the population goes extinct. In addition, as there are in total four traits evolving, all four traits need to obtain the right mutation, which is unlikely to happen in such a small population.

We found that there is selection to produce larger offspring and to decrease the body mass at metamorphosis in case the food source that larvae crucially depend on deteriorates. Larger offspring require less food to reach the metamorphosis size threshold and have therefore an advantage when the larval food source is scarce. In case adults are able to produce large enough offspring to skip this primary food source, direct development can evolve to avoid the dependence on the declining food source. It has often been observed in marine invertebrates (e.g., Marshall et al. 2012) and amphibians (e.g., Callery et al. 2001) that direct developing species produce larger eggs compared to related indirect developing species. Our phylogenetic analysis indeed supports our hypothesis that among amphibians the evolution of large eggs preceded the origin of direct development.

Here, we assumed a trade‐off between many small offspring that depend longer on the primary food source, and a few large offspring that metamorphose quickly after birth or even completely skip metamorphosis. Obviously, there are other benefits and disadvantages related to offspring size that we did not take into account. In marine invertebrates with external fertilization, for example, egg size affects fertilization success, with large eggs having a higher fertilization rate than small eggs in sperm‐limited environments, but small eggs having a fertilization advantage in sperm‐rich environments (Levitan 1993; Marshall and Keough 2007). For species with external fertilization, the evolution of direct development might be either impeded or facilitated by including size‐dependent fertilization success in our model. Mortality is often size dependent, with high mortality rates among the smallest individuals (e.g., Sogard 1997). When small individuals experience elevated mortality levels, there is probably a stronger selection to produce large offspring and direct development might evolve more easily. Offspring size will also affect dispersal ability in marine invertebrates (e.g., Marshall and Keough 2003). Large, nonfeeding, larvae, for example, have more time to disperse than small ones (Marshall and Keough 2003) and are more efficient swimmers (Wendt 2000). Feeding larvae spend in general more time in the plankton compared to nonfeeding larvae and can therefore disperse much further (Shanks et al. 2013). As fitness of sessile adults is affected by their dispersal potential as larvae (e.g., Marshall and Keough 2003), including dispersal in our model will likely affect the evolution of direct development.

Instead of producing larger offspring, individuals can also adapt to bad larval conditions by enhancing parental care, for example, by nursing their offspring, by supplying eggs with a large yolk reserve, or by providing larvae with nondeveloping nurse eggs, which provide nutrition during development. Providing larvae with nurse eggs allows mothers to increase their investment in their offspring, without facing the negative consequences of larger eggs. Although producing large eggs will likely reduce the larval period, it will at the same time increase the developmental time of the embryo (Marshall and Keough 2007; Maino et al. 2016; Marshall et al. 2018). Larger eggs are therefore exposed to egg mortality for a longer period, which might have consequences for the evolution of direct development. For further studies, it would be interesting to study how mortality during development affects the transition from metamorphosis to direct development and how this interacts with the evolution of nurse eggs. Parental care can greatly increase survival and growth rates of offspring and is therefore a good strategy when the larval food source is of poor quality. However, taking care of your offspring is energetically costly (e.g., Smith and Wootton 1995) and will reduce the number of offspring an individual can produce. Individuals will therefore face a similar trade‐off as is the case for producing larger offspring, they can either produce many offspring without taking care of them or produce a few and spend lots of energy in their upbringing. It is therefore likely that, as in the case for producing large offspring, the evolution of parental care depends on the conditions of the adult habitat. It has been shown in frogs that the evolution of large egg size typically precedes the evolution of parental care (Summers et al. 2006). Further work could address how parental care and producing large offspring interact with the evolution of direct development in case ecological conditions change.

In this study, we found that before direct development evolves, there is already selection to accelerate metamorphosis. Furthermore, we found that as soon as metamorphosis takes place before an individual is born, there is selection to completely get rid of the larval morphology. There is evidence for this last finding in the Calyptraeidae, a family of small marine gastropods. Collin (2004) showed with a phylogenetic framework that embryos of species that recently evolved direct development, closely resemble metamorphosing sister species. Embryos of species that evolved direct development early in evolutionary history, on the other hand, have highly modified embryos compared to metamorphosing sister species. Although our hypothesis that before direct development evolves metamorphosis occurs at an earlier age remains to be tested in a phylogenetic framework, there is some indirect empirical evidence for this finding. In sea urchins, for example, development of adult features is accelerated in direct developing species compared to metamorphosing species (Raff 1987). The direct developing Puerto Rican tree frog (*E. coqui*) has also accelerated the development of the adult morphology and has lost many of the larval structures (Elinson 2001).

We found that the body mass at which the secondary food source becomes available ($w_{\min}$) largely influences if direct development can evolve from metamorphosis or not. A metamorphosing population often goes extinct in case the secondary food source is available only for large individuals. Vice versa, when the secondary food source is already available for small individuals, direct development evolves easily. This finding might explain the high prevalence of direct developing species among marine invertebrates. Some marine invertebrates produce nonfeeding larvae that can already successfully complete metamorphosis coming from eggs smaller than 0.2 mm (which is about the width of a human hair) (Marshall et al. 2012; Falkner et al. 2015), indicating that for some species the adult food source is already available at a small size. Our results indicate that there is strong selection to change the body mass at which individuals have access to the secondary food source in case the primary food source becomes too scarce. Although including this trait ($w_{\min}$) in the evolutionary analysis will probably facilitate the evolution of direct development, there are often certain size limits to what a species can do with a specific morphology (Werner 1988) and therefore limits to which extent $w_{\min}$ can evolve. Piscivorous fish are, for example, limited by their gape size and need to be of a certain size before they are large enough to consume other fish (e.g., Mittelbach and Persson 1998). Furthermore, it is possible that the body mass at which the secondary food source becomes available has consequences for other life‐history traits. For example, when attack efficiencies decrease above a given body size (Persson et al. 1998), a secondary food source that is already available for small individuals might result in a smaller maximum adult body mass. Interestingly, in frogs terrestrial reproduction is associated with a reduction in adult body size (Gomez‐Mestre et al. 2012), which could indicate that direct development is indeed restricted to species which can switch to the secondary food source at a small size. For further research, it would be interesting to include a trade‐off between the body mass at which the secondary food source becomes available ($w_{\min}$) and other life‐history traits, to study how the evolution of $w_{\min}$ will affect the evolution of direct development.

In this article, we studied the evolutionary response of a metamorphosing population to deteriorating food conditions. However, there might be other factors than food driving the evolution of direct development, such as predation, interspecific competition, variation in environmental conditions, or hostile environments. Amphibians with direct development, for example, do no longer rely on water for reproduction (e.g., Elinson 2001), which can be a huge advantage in dry regions. Mortality rates often differ among habitats, which could strongly affect the loss of metamorphosis. Predation in the aquatic habitat is, for example, the main reason for blenny fish to move ashore for short periods of time (Ord et al. 2017). In marine invertebrates, the occurrence of direct developing species depends not only on mean food availability, but also on the seasonality and predictability of the environment (Marshall and Burgess 2015). Seasonal fluctuations in food conditions, for example, favor species with a dispersing, but nonfeeding, larval phase. Temperature might also affect the evolution of direct development because developmental time is negatively correlated to temperature (Gillooly et al. 2002; O'Connor et al. 2007). Therefore, there might be stronger selection to evolve direct development in colder environments in response to deteriorating food conditions. Indeed, data from marine invertebrates show that direct developers are more common in cold regions, whereas species with planktonic development occur more often in warm environments (Fernández et al. 2009; Marshall et al. 2012). In contrast to marine invertebrates, direct development seems to be largely confined to tropical regions in case of frogs (Gomez‐Mestre et al. 2012). One explanation for this observation could be that the aquatic environment in some tropical areas is harsh for newborn larvae due to oxygen limitation (Rollinson and Rowe 2018). Such harsh larval environments might select for a terrestrial lifestyle where individuals do no longer depend on the aquatic environment.

Although there are many possible biotic and abiotic conditions that could select for the evolution of direct development, in this article we focused on how food availability affects the evolutionary transition from metamorphosis to direct development. Even if other factors than food select for the evolution of direct development, food availability in both larval and adult habitat will have a strong effect on the larval period and on how much mothers can invest in their offspring. These life‐history traits in turn largely determine if direct development evolves or not. It is therefore crucial to understand how food conditions affect the evolutionary transition from metamorphosis to direct development. In addition, independent of the ecological driver of direct development, the results in this article show that it is not easy to evolve direct development because it requires high parental investment. We illustrate the importance of food in “Increased Mortality in the Larval Habitat” in the Appendix, where we study the response of a metamorphosing population to increased mortality rates in the larval habitat (e.g., due to predation, decreased oxygen availability, or other harsh conditions). We show that increased mortality rates in the larval habitat can facilitate the evolution of direct development, but ultimately food conditions determine if a metamorphosing population evolves direct development or not.

Among marine invertebrates and amphibians, there are many species with nonfeeding larvae that still undergo metamorphosis. Phylogenetic analyses of frogs show that this type of development is likely not an intermediate stage in the evolution of direct development (Gomez‐Mestre et al. 2012). Such strategies could, for example, be advantageous to allow for effective dispersal (in marine invertebrates), while at the same time not having to depend on an unreliable larval food source (Marshall and Burgess 2015). In our model, we did not allow for the evolution of such strategies. It would be interesting for further research to allow for the evolution of nonfeeding larvae and study how factors such as egg predation and dispersal affect the evolution of both direct development and nonfeeding larvae with metamorphosis.

Together, our results demonstrate that metamorphosis is a very successful strategy that is not easily lost. However, metamorphosis comes with a risk because it also makes individuals dependent on multiple food sources. An evolutionary response to changing conditions can prevent extinction, leading to a life‐history strategy with direct development. Direct development, however, can evolve only under limited conditions, leaving metamorphosing populations extremely vulnerable to habitat degradation.

## AUTHOR CONTRIBUTIONS

HTB and AMDR designed the study. REO designed and performed the phylogenetic approach. HTB performed the model analysis and wrote the first draft of the manuscript. All authors contributed to the final manuscript.

## DATA ARCHIVING

The code needed to reproduce the figures in the manuscript are deposited to Dryad (https://doi.org/10.5061/dryad.1g1jwstsc).

Associate Editor: O. Ronce

Handling Editor: M.R. Servedio

## Supporting information


**Table S1.1**. Model variables of the fat‐reserves model.
**Table S1.2**. Standard parameters of the fat‐reserves model.
**Table S1.3**. Parameters related to specialization and metamorphosis.
**Table S1.4**. Functions of the fat‐reserves model.
**Table S2.1**. Parameters of the generic size‐structured model.
**Table S3.1**. Differences and similarities between the two models'.
**Figure S5.1**. Density of the primary (panel A) and secondary (panel B) food source (mg L^‐1^), (C) population density (individuals per liter), and (D) age at metamorphosis (days) as a function of the supply rate of the primary food source (mg L^‐1^day^‐1^) in the presence (solid lines) and absence (dashed lines) of evolution for a species with metamorphosis. The black line in panel B represents the density of the secondary food source for a population with direct developers.
**Figure S5.2**. Density of the primary (panel A) and secondary (panel B) food source (mg L^‐1^), (C) population density (individuals per liter), and (D) age at metamorphosis (days) as a function of the supply rate of the primary food source (mg L^‐1^day^‐1^) in the presence (solid lines) and absence (dashed lines) of evolution for a species with metamorphosis.

## Figures and Tables

**Figure A1:**
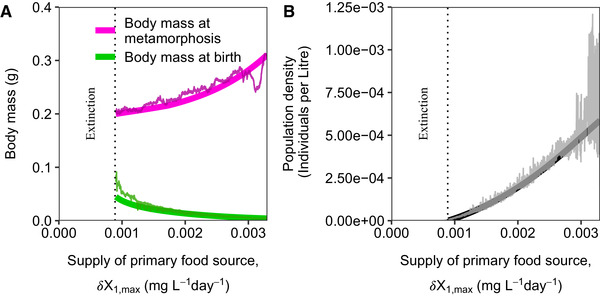
When the supply rate of the secondary food source is low, the population goes extinct in both deterministic model and stochastic IBM. The tick lines show the results from the deterministic model, the thin lines show the results of the IBM. For the IBM, we plot the mean trait value of the consumer population. (A) Body mass (g) at birth ($w_{b}$, light green) and body mass at metamorphosis ($w_{J}$, light purple), and (B) population density (number of individuals per liter) at the CSS for decreasing values of the supply rate of the primary food source ($\mathrm{mg}\;L^{-1}{\mathrm{day}}^{-1}$). The supply rate of the secondary food source equals $\delta X_{2},\max=0.0066\;\mathrm{mg}\;L^{-1}{\mathrm{day}}^{-1}$. For the IBM, the parameters equal υ=0.01, σ=0.01, $s=10^{8}$ L, and $\xi =10^{-11}\mathrm{mg}\;L^{-1}{\mathrm{day}}^{-1}$. Other parameter values are as shown in Tables S1.2, S1.3, and A2.

**Figure A2:**
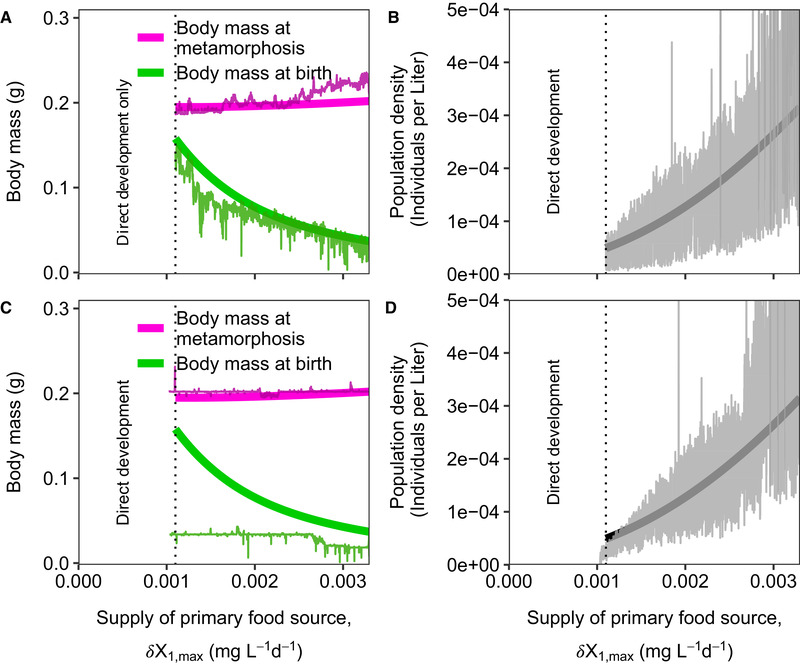
When the supply rate of the secondary food source is high, the population evolves direct development in the deterministic model. In the stochastic IBM the population either evolves direct development (upper panels and Fig. A3) or goes extinct (lower panels). The tick lines show the results from the deterministic model, the thin lines show the results of the IBM. We plot the mean trait value of the consumer population for the results of the IBM. (A, C) Body mass (g) at birth ($w_{b}$, green) and body mass at metamorphosis ($w_{J}$, light purple), and (B, D) population density (number of individuals per liter) at the CSS for decreasing values of the supply rate of the primary food source ($\mathrm{mg}\;L^{-1}{\mathrm{day}}^{-1}$). The supply rate of the secondary food source equals $\delta X_{2},\max=0.0165\;\mathrm{mg}\;L^{-1}{\mathrm{day}}^{-1}$. For the IBM, the parameters equal σ=0.01, $\xi =10^{-11}\mathrm{mg}\;L^{-1}{\mathrm{day}}^{-1}$, and $s=10^{7}$ L. For panels A and B the mutation rate υ equals = 0.01. For the lower panels C and D, υ=0.001. Other parameter values are as shown in Tables S1.2, S1.3, and A2.

**Figure A3:**
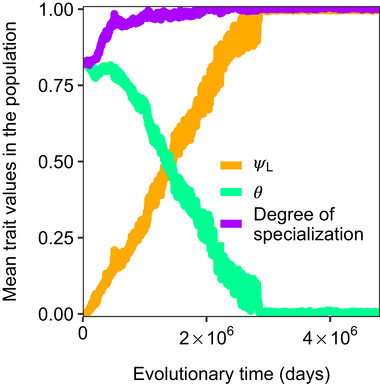
When the supply rate of the secondary food source is high, a metamorphosing population evolves sometimes direct development in the stochastic IBM for low values of the supply rate of the primary food source. Evolutionary dynamics, starting from a metamorphic initial population, the extent of metamorphosis (θ, mint green), the larval specialization parameter $\psi_{L}$ (orange), and the resulting specialization on the secondary food source for individuals with body mass $w\geq w_{J}$ (dark purple). We plot the mean trait value of the consumer population. The supply rate of the secondary food source equals $\delta X_{2},\max=0.0165\;\mathrm{mg}\;L^{-1}{\mathrm{day}}^{-1}$, that of the primary food source $\delta X_{1,\max}=0.0011\;\mathrm{mg}\;L^{-1}{\mathrm{day}}^{-1}$, which is the supply rate at which direct development can evolve (vertical dotted line in Fig. 2). The mutation rate υ equals = 0.01, σ=0.01, and $s=10^{8}$ L. Other parameter values are as shown in Tables S1.2, S1.3, and A2.

**Figure A4:**
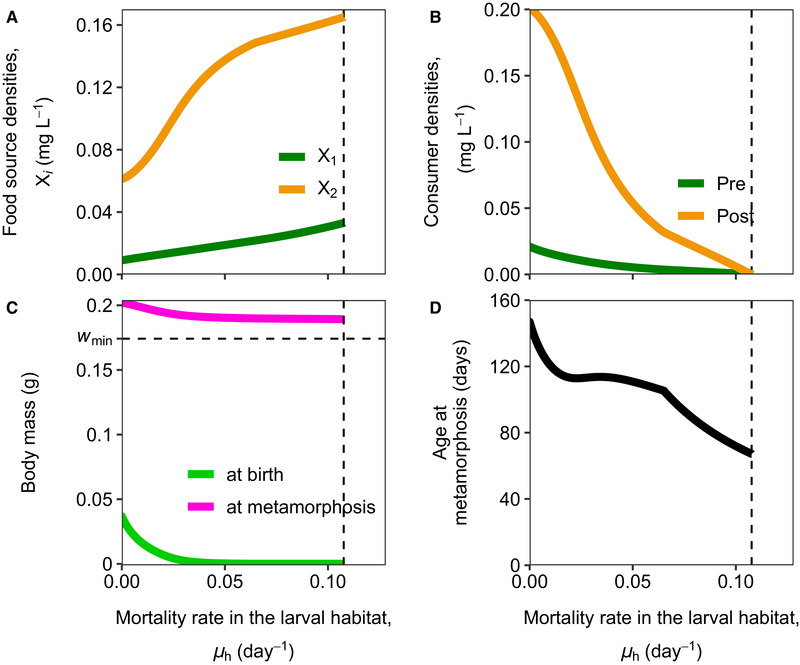
When the supply rates of both food sources are high, a metamorphosing population goes extinct when the mortality in the larval habitat is high (indicated with the vertical dotted lines). (A) Density of the food sources ($\mathrm{mg}\;L^{-1}$), (B) consumer densities ($\mathrm{mg}\;L^{-1}$), (C) body mass (g) at birth ($w_{b}$, green) and body mass at metamorphosis ($w_{J}$, light purple), and (D) age at metamorphosis at the ESSs as function of the additional mortality rate in the larval habitat (${\mathrm{day}}^{-1}$). The horizontal black dashed line in panel (C) indicates the body mass at which the secondary food source is available ($w_{\min}$). The dark‐green line in panel (B) represents the density of individuals before metamorphosis (with a body mass $w<w_{J}$), the orange line represents the density of individuals after metamorphosis. The supply rate of the primary food source equals $\delta X_{1},\max=0.0033\;\mathrm{mg}\;L^{-1}{\mathrm{day}}^{-1}$, the supply rate of the secondary food source equals $\delta X_{2},\max=0.0165\;\mathrm{mg}\;L^{-1}{\mathrm{day}}^{-1}$. Other parameter values are as shown in Tables S1.2 and S1.3.

**Figure A5:**
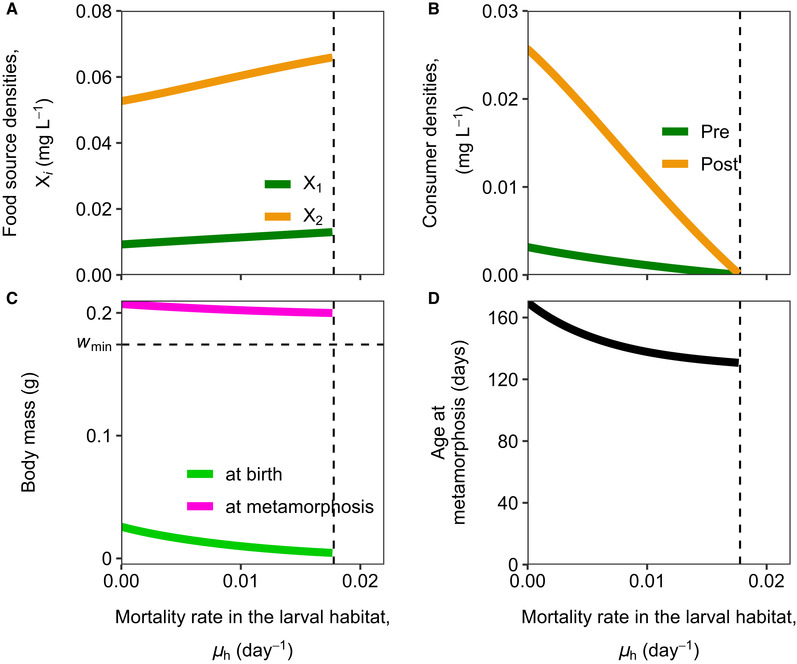
When the supply rates of both food sources are low, a metamorphosing population goes extinct when the mortality in the larval habitat is high (indicated with the vertical dotted lines). (A) Density of the food sources ($\mathrm{mg}\;L^{-1}$), (B) consumer densities ($\mathrm{mg}\;L^{-1}$), (C) body mass (g) at birth ($w_{b}$, green) and body mass at metamorphosis ($w_{J}$, light purple), and (D) age at metamorphosis at the ESSs as function of the additional mortality rate in the larval habitat (${\mathrm{day}}^{-1}$). The horizontal black dashed line in panel (C) indicates the body mass at which the secondary food source is available ($w_{\min}$). The dark‐green line in panel (B) represents the density of individuals before metamorphosis (with a body mass $w<w_{J}$), the orange line represents the density of individuals after metamorphosis. The supply rate of the primary food source equals $\delta X_{1},\max=0.0013\;\mathrm{mg}\;L^{-1}{\mathrm{day}}^{-1}$, the supply rate of the secondary food source equals $\delta X_{2},\max=0.0066\;\mathrm{mg}\;L^{-1}{\mathrm{day}}^{-1}$. Other parameter values are as shown in Tables S1.2 and S1.3.

**Figure A6:**
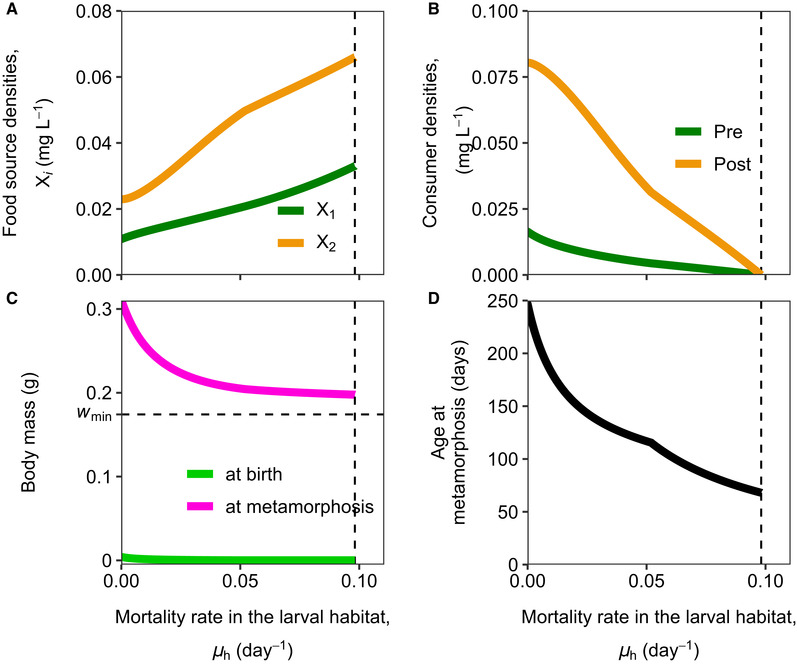
When the supply of the primary food source is high, and the supply rate of the secondary food source is low, a metamorphosing population goes extinct when the mortality in the larval habitat is high (indicated with the vertical dotted lines). (A) Density of the food sources ($\mathrm{mg}\;L^{-1}$), (B) consumer densities ($\mathrm{mg}\;L^{-1}$), (C) body mass (g) at birth ($w_{b}$, green) and body mass at metamorphosis ($w_{J}$, light purple), and (D) age at metamorphosis at the ESSs as function of the additional mortality rate in the larval habitat (${\mathrm{day}}^{-1}$). The horizontal black dashed line in panel (C) indicates the body mass at which the secondary food source is available ($w_{\min}$). The dark‐green line in panel (B) represents the density of individuals before metamorphosis (with a body mass $w<w_{J}$), the orange line represents the density of individuals after metamorphosis. The supply rate of the primary food source equals $\delta X_{1},\max=0.0033\;\mathrm{mg}\;L^{-1}{\mathrm{day}}^{-1}$, the supply rate of the secondary food source equals $\delta X_{2},\max=0.0066\;\mathrm{mg}\;L^{-1}{\mathrm{day}}^{-1}$. Other parameter values are as shown in Tables S1.2 and S1.3.

**Figure A7:**
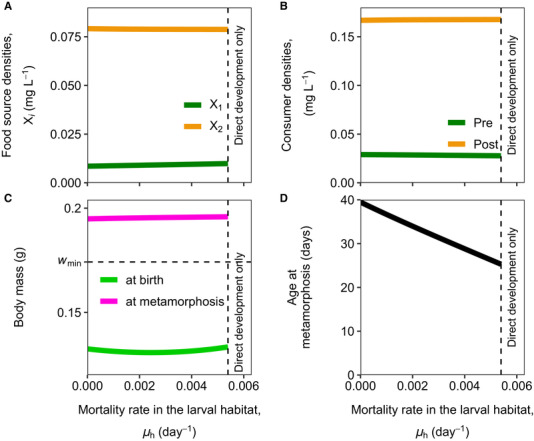
When the supply rate of the primary food source is low, and the supply rate of the secondary food source high, a metamorphosing population evolves direct development when the mortality in the larval habitat is high (indicated with the vertical dotted lines). (A) Density of the food sources ($\mathrm{mg}\;L^{-1}$), (B) consumer densities ($\mathrm{mg}\;L^{-1}$), (C) body mass (g) at birth ($w_{b}$, green) and body mass at metamorphosis ($w_{J}$, light purple), and (D) age at metamorphosis at the ESSs as function of the additional mortality rate in the larval habitat (${\mathrm{day}}^{-1}$). The horizontal black dashed line in panel (C) indicates the body mass at which the secondary food source is available ($w_{\min}$). The dark‐green line in panel (B) represents the density of individuals before metamorphosis (with a body mass $w<w_{J}$), the orange line represents the density of individuals after metamorphosis. For high mortality rates (at 0.0054 ${\mathrm{day}}^{-1}$), the life‐history strategy with metamorphosis becomes evolutionary unstable and is no longer an ESS. At this point, the population evolves to a life‐history strategy with direct development (see Fig. A8). The supply rate of the primary food source equals $\delta X_{1},\max=0.0013\;\mathrm{mg}\;L^{-1}{\mathrm{day}}^{-1}$, the supply rate of the secondary food source equals $\delta X_{2},\max=0.0165\;\mathrm{mg}\;L^{-1}{\mathrm{day}}^{-1}$. Other parameter values are as shown in Tables S1.2 and S1.3.

**Figure A8:**
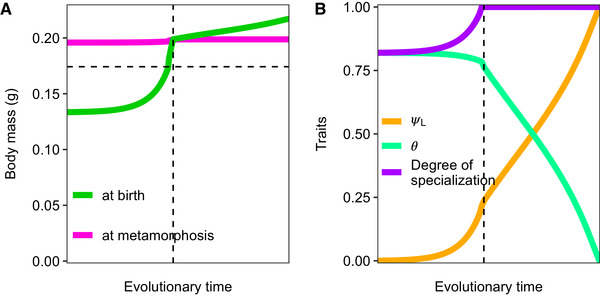
Evolutionary dynamics, starting from a metamorphic initial population, of (A) the body mass at birth ($w_{b}$, green) and at metamorphosis ($w_{J}$, light purple) in gram, and of (B) the extent of metamorphosis (θ, mint green), the larval specialization parameter $\psi_{L}$ (orange), and the resulting specialization on the secondary food source for individuals with body mass $w\geq w_{J}$ (dark purple). The vertical dashed line indicates the moment where direct development evolves via internalization (panel C in Fig. 1). The supply rate of the secondary food source equals $\delta X_{2},\max=0.0165\;\mathrm{mg}\;L^{-1}{\mathrm{day}}^{-1}$, that of the primary food source $\delta X_{1,\max}=0.0013\;\mathrm{mg}\;L^{-1}{\mathrm{day}}^{-1}$. The additional mortality rate in the larval habitat equals $\mu_{h}=0.0054\;{\mathrm{day}}^{-1}$, which is the mortality rate at which direct development can evolve (vertical dotted lines in Fig. A4). The horizontal dashed line in panel (A) indicates the body mass at which the secondary food source is available ($w_{\min}$). Other parameter values are as shown in Tables S1.2 and S1.3.

**Figure A9:**
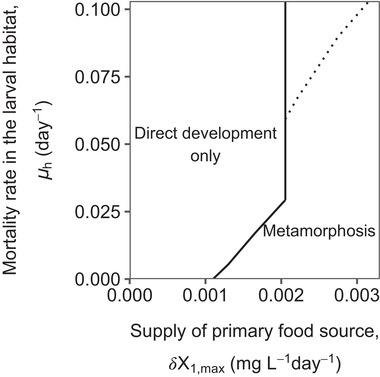
Only for low supply rates of the primary food source can habitat‐specific mortality select for the evolution of direct development. For additional mortality rates above the black line, a metamorphosing population will evolve direct development. For high supply rates of the primary food source (on the right side of the black line), a metamorphosing population will go extinct when the additional mortality in the larval habitat becomes too high (dotted line). The supply rate of the secondary food source equals $\delta X_{2},\max=0.0165\;\mathrm{mg}\;L^{-1}{\mathrm{day}}^{-1}$. Other parameter values are as shown in Tables S1.2 and S1.3.

**Figure A10:**
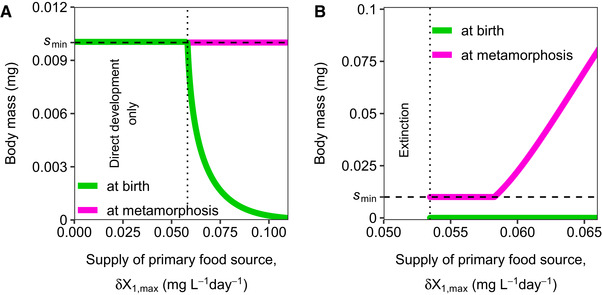
The evolutionary response of a metamorphosing population for decreasing values of the supply rate of the primary food source. Body size (mg) at birth ($s_{b}$, green) and body size at metamorphosis ($s_{j}$, light purple). (A) When the supply rate of the secondary food source is high, a metamorphosing population evolves direct development. The supply rate of the secondary food source equals 0.08 mg L${}^{-1}$ day${}^{-1}$. (B) When the supply rate of the secondary food source is low, a metamorphosing population goes extinct for low supply rates of the primary food source. The supply rate of the secondary food source equals 0.055 mg L${}^{-1}$ day${}^{-1}$. The body weight at which the secondary food source becomes available equals $x_{\min}=0.01$ mg in both panels. Other parameter values are shown in Table S2.1.

**Figure A11:**
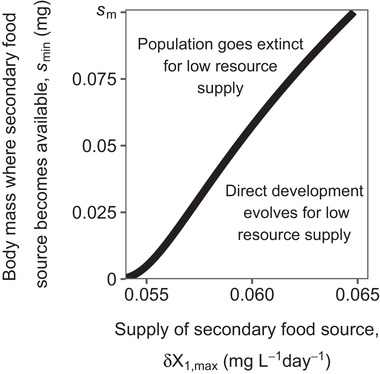
The smaller the body size where the secondary food source is available ($s_{\min}$), the easier it is to evolve direct development. Two‐parameter plot showing where direct development can evolve. The horizontal axis shows the supply rate of the secondary food source ($\delta X_{2},\max(\;\mathrm{mg}\;L^{-1}{\mathrm{day}}^{-1}$)), the vertical axis shows the body size at which this food source becomes available ($s_{\min}$ (g)). Below the black line, direct development can evolve for low supply rates of the primary food source. A metamorphosing population goes extinct when the supply rate of the primary food source decreases for parameter combinations above the black line. Parameter values are as shown in Table S2.1.

**Figure A12:**
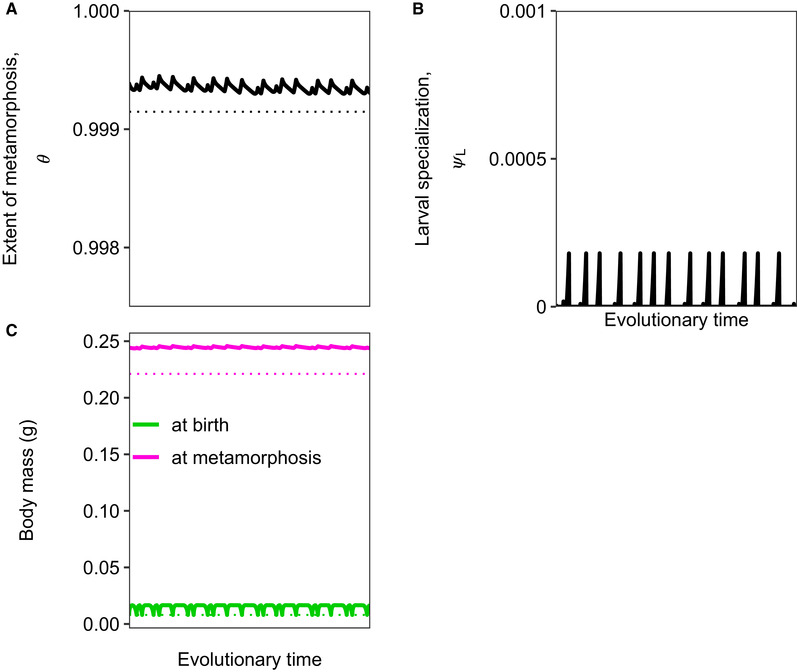
Evolutionary dynamics of the extent of metamorphosis θ (A), the larval specialization parameter $\psi_{L}$ (B), and the body mass at birth $w_{b}$ and metamorphosis $w_{J}$ (C) in case the life‐history strategy for which the selection gradient vanishes corresponds to an unstable ecological equilibrium. The dotted lines indicate the value of the trait in the ESS in the ecologically (and hence evolutionary) unstable equilibrium. $\delta X_{1,\max}=0.0055$ and $\delta X_{2,\max}=0.0165\;\mathrm{mg}\;L^{-1}{\mathrm{day}}^{-1}$. Other parameters are as shown in Tables S1.2 and S1.3.

## Appendix 1

### LOG MARGINAL LIKELIHOODS

In this Appendix, we show the log marginal likelihoods for the three different models. These indicate very strong support for the dependent model over the independent model, as well as support for the dependent model over the dependent constrained model.

### RESULTS OF THE INDIVIDUAL‐BASED MODEL

The description and the update rules of the individual‐based model (IBM) can be found in Methods S4. Here, we show the results of the simulations.

The results from the IBM confirm the results from the deterministic model. With decreasing supply rates of the primary food source, the body mass at metamorphosis decreases whereas the body mass at birth increases (Figs. A1 and A2). In the deterministic model, we found that for low supply rates of the secondary food source, a metamorphosing population goes extinct for diminishing supply rates of the primary food source. Not surprisingly, we find a similar result for the IBM (Fig. A1). The consumer population always goes extinct, independent of mutation rate υ, standard deviation of the mutational step size σ, size of the system *s*, or speed of habitat degradation ξ.

In the deterministic model, direct development evolves from metamorphosis for high supply rates of the secondary food source. In the IBM, direct development evolves in almost two‐thirds of our simulations (39 out of 64, see upper panels in Figs. A2 and A3, for example), whereas the population goes extinct in the rest of our simulations (see lower panels in Fig. A2, for example). Direct development is more likely to evolve when the size of the system *s* is large, when the mutation probability υ is high, when the mutational step size is large (high value for standard deviation σ), and when the speed of habitat degradation ξ is low. A large size of the system allows for a larger consumer population, which decreases the risk of extinction due to stochasticity. A high mutation probability, large mutational step size, and slow speed of habitat degradation, all allow the population to more easily track the change in environmental conditions, thereby increasing the probability for direct development to evolve. Vice versa, with a low mutation probability, small mutational step size, and fast habitat degradation, it is much harder for the population to adapt quickly enough to changing environmental conditions. In the example shown in the lower panels in Figure A2, the population was not able to evolve large enough offspring to deal with the decrease in primary food supply, leading to the extinction of the population. The results of the IBM simulations in case of a high supply rate of the secondary food source are summarized in Table A3.

**Table A1:** Log marginal likelihood for the dependent, independent, and constrained dependent models obtained from Markov chain Monte Carlo (MCMC) in BayesTraits, for the evolution of direct development and large egg size on the amphibian phylogeny

			Constrained
	Dependent	Independent	Dependent
	Model	Model	Model
MCMC 1	−599.73	−617.87	−605.72
MCMC 2	−608.28	−617.16	−606.12
MCMC 3	−605.11	−615.73	−607.32
MCMC 4	−607.58	−615.96	−607.48
MCMC 5	−605.82	−614.42	−607.35
Average log marginal	−605.3	−616.23	−607.8
likelihood			
Log Bayes factor	21.85		
(dependent vs. independent)			
Log Bayes factor	2.99		
(dependent vs. constrained dependent			
P(D = 0 →1 | E = 0) = P(D = 0 →1 | E = 1))			

*Note*: For each of the models, five independent MCMC chains were run for 5,000,000 generations, discarding 10% as burn‐in . The average log marginal likelihoods for the dependent, independent, and constrained dependent models were used to calculate a log Bayes factor.

**Table A2:** Parameters of the IBM

Parameter	Description	Values	Unit
$q_{s}$	Proportionality constant determining at which level of reversible mass starvation mortality starts	0.2	–
λ	Constant for starvation mortality	0.2	day${}^{-}1$
υ	Mutation probability	0.001 or 0.01	–
σ	Standard deviation of the	0.01 or 0.1	gram for $x_{b}$ and $x_{J}$,
	Mutation distribution		dimensionless for $\psi_{L}$ and θ.
ξ	Speed of habitat deterioration	$10^{-11}$ or $10^{-10}$	$\mathrm{mg}\;L^{-1}$ day${}^{-1}$
*s*	Size of the system	$10^{7}$ or $10^{8}$	Liters

**Table A3:** Summary of IBM simulations in case of a high supply rate of the secondary food source $\delta X_{2},\max$. A total of 64 simulations were run

Parameter	High/Fast (%)	Low/Slow (%)
Speed of habitat deterioration (ξ)	43.75	78.13
Mutation probability (υ)	84.38	37.5
Standard deviation of the mutation distribution (σ)	71.88	50
Size of the system (*s*)	81.25	40.6

*Note*: The numbers in the table show the percentage of runs that resulted in the evolution of direct development . The supply rate of the secondary food source equals $\delta X_{2},\max=0.0165\;\mathrm{mg}\;L^{-1}{\mathrm{day}}^{-1}$. Other parameter values are as shown in Tables S1.2, S1.3, and 4A2

To conclude, our simulations show that relaxing the assumptions of adaptive dynamics do not affect our main conclusions.

### INCREASED MORTALITY IN THE LARVAL HABITAT

Here, we show how a metamorphosing population responds to increased mortality rates in the larval habitat. We assume that the more time individuals forage on the primary food source, the higher the mortality rate they are exposed to. Therefore, the size‐specific mortality rate individuals experience equals

(C1) $$\mu \left(w,X_{1},X_{2}\right)=\mu_{b}+\mu_{h}\phi \left(w,X_{1},X_{2}\right),$$

where parameter $\mu_{h}$ determines the maximum habitat‐specific mortality rate and $\phi (w,X_{1},X_{2})$ indicates the relative preference for the primary food source (see Methods S1). As small individuals ($w<w_{\min}$) feed on the primary food source only, they always experience the maximum habitat‐specific mortality rate $\mu_{h}$. Large individuals, vice versa, mainly feed upon the secondary food source and are therefore hardly exposed to this additional mortality.

We show the evolutionary response for a case where the supply rates of both food sources are high (Fig. A4), for a case where the supply rates of both food sources are low (Fig. A5), for a case with a low supply rate of the secondary food source and a high supply rate of the primary food source (Fig. A6), and for a case with a low supply rate of the primary food source and a high supply rate of the secondary food source (Fig. A7). Only in the latter case direct development evolves (Fig. A8).

Increasing the mortality rate in the larval habitat decreases the density of the population (panel B in Figs. A4, A5, A6, A7). This decrease in the consumer density relaxes the competition for food, resulting in an increase in the density of the primary food source (panel A in Figs. A4, A5, A6, A7). The increase in the primary food source leads to faster growth and an earlier age at maturation (panel D in Figs. A4, A5, A6, A7). Increasing the mortality in the larval habitat selects for a smaller body mass at metamorphosis (panel C in Figs. A4, A5, A6, A7). The high density of the primary food source allows larvae to grow fast and metamorphose early in life. Therefore, there is, counterintuitively, initially selection to produce many small individuals, rather than a few big ones.

When the supply rate of the primary food source is high or the supply rate of the secondary food source low, increasing mortality in the larval habitat always leads to the extinction of the metamorphosing populations (panel B in Figs. A4, A5, A6). Because of the high availability of the primary food source, there is never selection to produce larger offspring (panel C in Figs. A4, A5, A6), even not when the mortality experienced by larvae is high. Ultimately, the evolutionary attractor of the metamorphosing population collides with its extinction boundary for high mortality rates and the population goes extinct.

When the supply rate of the primary food source is low and the supply rate of the secondary food source high, for high mortality rates the increase in the primary food source no longer compensates for the increase in mortality. Hence, the direction of selection reverses for high habitat‐specific mortality, such that individuals will produce larger offspring instead of smaller (Fig. A7C). As the supply rate of the secondary food source is high, adults are able to produce large larvae that rely on the primary food source only for a short period. When the primary food source becomes scares, there is therefore selection to specialize on the secondary food source, which will ultimately lead to the evolution of direct development (Figs. A7 and A8).

In Figure A9 we show a two‐parameter plot indicating for which combinations of supply rates of the primary food source and habitat‐specific mortality rates a metamorphosing population evolves direct development. We show this plot only for a high supply of the secondary food source because for low supply rates of this food source direct development never evolves. Figure A9 shows that increased mortality in the larval habitat selects for the evolution of direct development only when the supply rate of the primary food source is low. In case the supply rate is high, a metamorphosing population will go extinct when the mortality in the larval habitat becomes too high.

In summary, when the larval habitat deteriorates due to increased mortality rates, direct development can evolve only under specific food conditions. These food conditions are qualitatively similar as in the absence of this habitat‐specific mortality: Direct development evolves hence for high supply rates of the secondary food source and low supply rates of the primary food source.

### GENERALITY OF RESULTS

In this Appendix, we study the evolution of direct development from metamorphosis in a generic size‐structured population model, based on the model described in de Roos and Persson (2013, ch. 9). The model description and default parameters can be found in Methods S2. In Table S3.1, we give an overview of the differences and similarities of the fat‐reserves model and the generic model. Here, we show that many of our results are robust against substantial differences in model structure and parameters.

When the supply rate of the primary food source diminishes, there is an evolutionary response to reduce the period individuals spend feeding upon this food source (Fig. A10). Depending on the supply rate of the secondary food source, this either results in the evolution of direct development (panel A in Fig. A10), or in the extinction of the population (panel B in Fig. A10). As before, we find that the earlier individuals have access to the secondary food source (small values of $s_{\min}$), the easier direct development can evolve (Fig. A11).

When the secondary food source is in high supply, the optimal size at birth increases with a decrease in the supply rate of the primary food source (Fig. A10A). When the primary food source is in low enough supply (vertical line in Fig. A10A), adults produce offspring with a body size larger than the body size at metamorphosis. Individuals do therefore no longer undergo metamorphosis and direct development has evolved. As in the main text, this life‐history strategy with direct development is also present for high supply rates of the primary food source (for clarity not shown in the figure).

For low supply rates of the secondary food source, there is an evolutionary response to metamorphose at a smaller body size with a decreasing supply rate of the primary food source (Fig. A10B). For low supply rates of the primary food source, the metamorphosing population goes extinct (vertical line in Fig. A10B). In contrast to the model analyzed in the main text, there is not an alternative life‐history strategy present where individuals have direct development.

### EVOLUTIONARY CYCLING

Here, we show how the four traits evolve in case there is no stable evolutionary endpoint.

For high values of the primary food source, the ESS is often absent. The reason for this is that there are two stable ecological equilibria, separated by an unstable equilibrium. The life‐history strategy for which the selection gradient equals 0 is in this case located on the ecologically unstable equilibrium branch and can therefore never be reached (we will refer to this particular life‐history strategy as an unstable ESS, even though this is in the context of adaptive dynamics somewhat of a misnomer). In each of the two stable equilibrium states evolution takes the evolving strategies to the boundary of the existence of this equilibrium, at which point the system switches to the other ecological stable equilibrium. As a consequence, the four traits keep on changing over evolutionary time. As there is no stable ESS occurring for these parameter values, the traits stay relatively close to the trait values that characterize the unstable ESS (Fig. A12).

Note that the evolving traits, except for specialization parameter $\psi_{L}$, fluctuate around a value shifted away from the ESS. In case of only a single evolving trait, the evolving trait would fluctuate around the (unstable) ESS, where the direction of evolution changes each time the trait has reached a limit point. However, because in our model four traits can evolve, a four‐dimensional trait space makes these cyclic dynamics more complex. Due to evolution in the other traits, the limit points of a focal trait change over evolutionary time, which will also change the parameter range over which this trait will cycle. Therefore, only a single trait fluctuates around its ESS value, thereby changing the parameter range over which the other three traits cycle. Here, this happens for the specialization parameter $\psi_{L}$. However, as this value cannot reach values below zero (which is its minimum value), it will fluctuate between the ESS value and a value slightly above zero.
