# Supplementary material for: Habitat deterioration promotes the evolution of direct development in metamorphosing species
Source: Evolution. 2020 Jun 24;74(8):1826–50. doi: 10.1111/evo.14040 (PMC7496874; doi:10.1111/evo.14040)
Supplement: Supplementary file 1 — Table S1.1. Model variables of the fat‐reserves model. Table S1.2. Standard parameters of the fat‐reserves model. Table S1.3. Parameters related to specialization and metamorphosis. Table S1.4. Functions of the fat‐reserves model. Table S2.1. Parameters of the generic size‐structured model. Table S3.1. Differences and similarities between the two models'. Figure S5.1. Density of the primary (panel A) and secondary (panel B) food source (mg L‐1), (C) population density (individuals per liter), and (D) age at metamorphosis (days) as a function of the supply rate of the primary food source (mg L‐1day‐1) in the presence (solid lines) and absence (dashed lines) of evolution for a species with metamorphosis. The black line in panel B represents the density of the secondary food source for a population with direct developers. Figure S5.2. Density of the primary (panel A) and secondary (panel B) food source (mg L‐1), (C) population density (individuals per liter), and (D) age at metamorphosis (days) as a function of the supply rate of the primary food source (mg L‐1day‐1) in the presence (solid lines) and absence (dashed lines) of evolution for a species with metamorphosis. [file EVO-74-1826-s001.pdf]

Methods S1

Model description of the fat-reserves model

In this section we describe the size-structured model as already presented in ten Brink et al. (2019). We changed the energetic costs of metamorphosis in comparison with the model in ten Brink et al. 2019 to ensure individuals do not become smaller after metamorphosis than their size at birth. This would happen in case the irreversible mass at metamorphosis, x_J, is close to the irreversible body mass at birth x_b. In the paper of ten Brink et al. (2019), the body mass at birth did not evolve and we therefore never encountered such a situation. A quick analysis showed that changing the energetic costs of metamorphosis does not affect the results of ten Brink et al. (2019) (results not shown).

We assume that there are two, unstructured, food sources present. Both the primary and secondary food source follow semi-chemostat dynamics with turnover rate δ, and will reach, in the absence of consumers, a density of $X_{1,\max}$ and $X_{2,\max}$ respectively. The primary food source, with density X_1, is available for all individuals while the secondary food source, with density X_2, is only available for large individuals. The two food sources require two different morphologies to be efficiently utilised by the consumers.

We assume that a consumer consists of two different forms of mass, irreversible mass x such as bones and organs and reversible mass y such as fat. The reversible mass of an individual can be invested in metamorphosis or used to cover its basic metabolism under starvation conditions. For simplicity we assume equilibrium conditions and ignore starvation conditions; an individual's reversible mass is therefore fully available for covering the costs of metamorphosis. The body length, attack rate, and handling time of an individual depend only on its standardised body mass $w = x + y_{\max} = x(1+q_J)$, where $y_{\max} = q_J x$ is the maximum attainable amount of reversible body mass. Parameter q_J is a dimensionless scaling constant describing an individual's maximum ratio of reversible to irreversible mass.

Table S1.1: Model variables of the fat-reserves model

Variable	Description	Range	Unit
X_1	Density of primary food source	From 0 to $X_{1,\max}$	mg L^{-1}
X_2	Density of secondary food source	From 0 to $X_{2,\max}$	mg L^{-1}
x	Irreversible body mass	Larger than x_b	g
y	Reversible body mass	From $q_J x_b$ to $y_{\max}$	g

Newborn larvae (L) are born at an irreversible body mass x_b and the maximum attainable amount of reversible mass $y = q_J x_b$. The total body mass at birth therefore equals $w_b = (1+q_J)x_b$. The ratio between irreversible and reversible mass is constant until individuals reach standardised body mass w_J and metamorphose into juveniles. Individuals lose an amount $\theta(x_J - x_b)(q_J - q_m)$ of their reversible body mass during metamorphosis. In this equation θ is the extent of the meta-

morphosis and parameter q_{m} is the ratio of y over x of an individual immediately after full metamorphosis ($\theta = 1$). In contrast to ten Brink et al. 2019, we assume that individuals can invest only reversible mass they gained after birth ($(x_{\mathrm{J}} - x_{\mathrm{b}})q_{\mathrm{J}}$) into metamorphosis. This assumption implies that there are no energetic costs to metamorphosis in case it takes place before birth.

After metamorphosis the reversible body mass y is over time restored to $y_{\max} = q_{\mathrm{J}}x$ (see below) such that the total body mass $x + y$ again equals its standardised body mass. We therefore use the term body mass to refer to the standardised body mass w. Juveniles mature into adults (A) and start reproducing when reaching standardised body mass w_{A}. The secondary food source X_2 becomes available after individuals have reached standardised body mass $w_{\min}$.

The size-dependent attack rates on the two food sources are described by two hump-shaped functions following

$$a_1(w) = A_{1i}[\frac{w}{w_0}\exp(1 - \frac{w}{w_0})]^{\alpha} \tag{S1.1a}$$

$$a_2(w) = \begin{cases} 0 & w \leq w_{\min} \\ A_{2i}[\frac{w - w_{\min}}{w_0}\exp(1 - \frac{w - w_{\min}}{w_0})]^{\alpha} & \text{otherwise.} \end{cases} \tag{S1.1b}$$

In these equations α determines how strongly the attack rates increase and decrease around the peaks at w_0 and $w_0 + w_{\min}$, respectively. Parameters A_{1i} and A_{2i} are the maximum attack rates an individual can reach on the primary and secondary food source when its standardised body mass equals w_0 and $w_0 + w_{\min}$, respectively. We assume that there is a linear trade-off between these two maximum attack-rate constants within a certain life stage (i = L, J, or A),

$$\begin{aligned} A_{1i} &= (1 - \psi_i)(A_{\max} - A_{\min}) + A_{\min}, \\ A_{2i} &= \psi_i(A_{\max} - A_{\min}) + A_{\min}. \end{aligned} \tag{S1.2}$$

In these equations, $0 \leq \psi_i \leq 1$ is the relative degree of specialisation on the secondary food source of a certain life stage. A value of $\psi_i = 0$ means that individuals in life stage i are completely specialised in feeding on the primary food source and not very efficient in feeding on the secondary food source. Vice versa, a value of $\psi_i = 1$ means that individuals are very efficient in feeding on the secondary food source while they are not very efficient in feeding on the primary food source.

Metamorphosis decouples the different life stages such that individuals can be specialised on the primary food source as larvae and on the secondary food source as juveniles and adults. Metamorphosis decouples the different life stages as follows

$$\psi_{\mathrm{A}} = \psi_{\mathrm{J}} = \min(1, \psi_{\mathrm{L}} + \theta), \tag{S1.3}$$

in this equation parameter θ is the extent of the metamorphosis. Individuals that undergo metamorphosis lose part of their body mass as described above and furthermore have a probability of $\rho\theta$ to die during metamorphosis.

The food intake of an individual with standardised body mass w can be written as

$$I(w,X_1,X_2) = \frac{\phi(w,X_1,X_2)a_1(w)X_1 + [1-\phi(w,X_1,X_2)]a_2(w)X_2}{1+h(w)\{\phi(w,X_1,X_2)a_1(w)X_1 + [1-\phi(w,X_1,X_2)]a_2(w)X_2\}} \tag{S1.4a}$$

whereby the handling time $h(w)$ equals (following Persson et al. 1998)

$$h(w) = \zeta_1 + \zeta_2 w^{-\zeta_3} \mathrm{e}^{\zeta_4 w}. \tag{S1.4b}$$

We assume that individuals have a relative preference of $\phi(w,X_1,X_2)$ for the primary food source, where $\phi(w,X_1,X_2)$ equals

$$\phi(w,X_1,X_2) = \frac{1}{1+\mathrm{e}^{\varsigma(a_2(w)X_2 - a_1(w)X_1)}}. \tag{S1.5}$$

In this equation parameter ς determines the steepness of the sigmoid, food-selection curve at equal food source profitabilities, $a_1(w)X_1 = a_2(w)X_2$ (de Roos et al. 2002). The form of equations S1.1 and S1.5 imply that large individuals ($w > w_{\mathrm{min}}$) always include both food sources in their diet. We will, however, for convenience state that individuals only feed upon the primary or secondary food source in case the fraction of the secondary food source in the diet of large individuals is graphically indistinguishable from 0 or 1, respectively.

The total energy-intake of an individual equals its food-intake rate multiplied by a conversion factor κ_{e}. Total net-energy intake is first used to cover maintenance costs. The metabolic demands per unit of time is a function of both irreversible and reversible mass of a consumer and can be described by a power function following

$$E_{\mathrm{m}}(x,y) = p_1(x+y)^{p_2}. \tag{S1.6}$$

Larvae and juveniles allocate a fraction $\kappa_{\mathrm{J}}(x,y)$ of the net-biomass production (the difference between the food assimilation and maintenance cost of an individual) $E_{\mathrm{g}}(x,y,X_1,X_2) = k_{\mathrm{e}}I(w,X_1,X_2) - E_{\mathrm{m}}(x,y)$ to growth in irreversible mass, following

$$\kappa_{\mathrm{J}}(x,y) = \frac{y}{(1+q_{\mathrm{J}})q_{\mathrm{J}}x}. \tag{S1.7a}$$

The remaining part is allocated to growth in reversible mass. Since adults also invest in reproduction they allocate a lower fraction $\kappa_{\mathrm{A}}(x,y)$ to growth in irreversible mass following

$$\kappa_{\mathrm{A}}(x,y) = \frac{y}{(1+q_{\mathrm{A}})q_{\mathrm{A}}x}, \tag{S1.7b}$$

with $q_{\mathrm{A}} > q_{\mathrm{J}}$, the remainder is invested in reversible mass and reproduction. To ensure that individuals will aways invest in reversible mass in such a way that the ratio of y to x either remains or is restored to q_{J} and that reproduction does not take place when $y < q_{\mathrm{J}}x$ (Persson et al. 1998)

we assume that adults invest a fraction $\kappa_R(x,y)$ of their net-energy production in reversible mass according to the function

$$\kappa_R(x,y) = \begin{cases} 1 - \kappa_A(x,y) & y < q_J x \\ (1 - \kappa_J(x,y))\frac{\kappa_A(x,y)}{\kappa_J(x,y)} & \text{otherwise.} \end{cases} \tag{S1.8}$$

The remainder fraction of the adult net-biomass production $(1 - \kappa_A(x,y) - \kappa_R(x,y))$ is invested in reproduction. The number of eggs an individual adult produces per unit of time then equals

$$b(x,y,X_1,X_2) = \begin{cases} 0 & y < q_J x \\ (1 - \frac{\kappa_A(x,y)}{\kappa_J(x,y)})E_g(x,y,X_1,X_2)\eta/w_b & \text{otherwise.} \end{cases} \tag{S1.9}$$

where η is a conversion factor.

All individuals have a per capita background mortality rate of μ_b.

Direct development

We assume that in case metamorphosis takes place before individuals are born ($w_J < w_b$), the costs of metamorphosis are subsumed into the costs for the mother to produce a single offspring. This means that a fraction of the eggs ($\rho\theta$) does not survive. We assume that the energetic costs of metamorphosis are negligible in case metamorphosis takes place before birth and that eggs do not lose mass during development. The number of eggs an individual adults produces per unit of time therefore equals

$$b(x,y,X_1,X_2) = \begin{cases} 0 & y < q_J x \\ (1-\rho\theta)(1 - \frac{\kappa_A(x,y)}{\kappa_J(x,y)})E_g(x,y,X_1,X_2)\eta/w_b & \text{otherwise,} \end{cases} \tag{S1.10}$$

in case metamorphosis takes place before individuals are born. It is likely that metamorphosis is less costly in case the mothers pay for it. Predation risk, for example, is relatively high during metamorphosis for free-living individuals (e.g., Wassersug and Sperry 1977), but this is unlikely to be case when metamorphosis takes place before birth. However, assuming that metamorphosis is less costly for mothers does not change the results (not shown). Metamorphosis is no longer beneficial when it takes place before birth, since all free-living individuals will have the same (adult) morphology. A small cost of metamorphosis will therefore already result in selection to completely get rid of metamorphosis (i.e., θ will evolve to zero, see for example figure 3B).

Metabolic demands and handling time are parameterised for the interaction between a planktivorous fish population of roach *Rutilus rutilus* and two zooplankton populations as food sources following Persson et al. (1998). The model should, however, be interpreted as a more general consumer-resource model describing the interaction between two food sources and a size-structured consumer. All rates were scaled to a daily basis, all parameter values related to energetics are based on a reference temperature of 19°C. Processes taking place during the winter season are ignored. We assume that eggs have a minimal mass of 1 10^{-4} gram, which is the smallest egg

size observed for cold-blooded aquatic invertebrates (Hendriks and Mulder 2008). Model variables are listed in table S1.1, the evolving parameters in table 1 and standard parameter values in tables S1.2 and S1.3.

Evolutionary dynamics

To study under which conditions metamorphosis disappears we use the framework of adaptive dynamics (Dieckmann and Law 1996; Geritz et al. 1998). Adaptive dynamics assumes that a population is monomorphic and that evolution in this population occurs because of the fixation of small and rare mutations. The extent of metamorphosis θ, specialisation parameter ψ_L, the body mass at birth w_b and the body mass at which metamorphosis takes place w_J can all evolve. For the latter two we assume that the amount of irreversible mass at metamorphosis (x_J) and birth (x_b) evolve while parameter q_J, that determines together with the irreversible mass x the total body mass, does not evolve. We assume that initially both supply rates are high ($\delta X_{1,\max} = \delta X_{2,\max} = 0.011\text{mg L}^{-1}\text{day}^{-1}$) and individuals undergo metamorphosis ($\theta > 0$). In this case there is only a single evolutionary singular strategy (ESS), which is defined by the vanishing of the selection gradient of all considered traits. We track this ESS for different values of $w_{\min}$ as a function of the supply rate of the primary food source and determine whether these strategies are convergence stable and/or evolutionary stable following Geritz et al. (1998) and Leimar (2009).

We used the PSPManalysis software package (de Roos 2016) to analyse our model. The PSPManalysis package numerically calculates the ecological equilibrium of our model as a function of any model parameter, by iteratively computing the food densities for which the lifetime reproductive success R_0 of an individuals equals 1. In our model, R_0 depends on the size-specific rates of feeding, growth, mortality, and fecundity of the consumer, the PSPManalysis package therefore numerically integrates a set of coupled ordinary differential equations that describe how these size-specific rates change of the lifetime of an individual. The package automatically detects evolutionary singular strategies and can numerically continue these ESSs as a function of any second model parameter.

Population-level model

Here, we describe the model equations, defining the system at the population level. All functions of the model are listed in table S1.4. In principle, the population state would be described by a density function $n(t,x,y)$, representing the density of individuals with irreversible mass x and reversible mass y at time t (Metz and Diekmann 1986). However, formulating a partial differential equation (PDE) for the density function $n(t,x,y)$ leads to mathematical difficulties. The reason for this is that the individual state space is two-dimensional, spanned by irreversible mass x and reversible mass y, but that the support of the density function $n(t,x,y)$ is only one-dimensional. Since all individuals are born with the same state at birth, individuals that are born at the same moment in time will always have the same values of x and y throughout their life. As a consequence, the density function $n(t,x,y)$ adopts nonzero values only at the one-dimensional curve that represents the unique relationship between x and y and is 0 for all other values of x and y. This also implies that the density function $n(t,x,y)$ is non-differentiable in its last two arguments, as it

jumps discontinuously from its nonzero value at its one-dimensional support to 0 for all other values. For this reason, partial derivatives like $\partial n(t,x,y)/\partial x$ and $\partial n(t,x,y)/\partial y$ that would occur in a PDE for $n(t,x,y)$ are mathematically ill defined. To cope with this singularity, the model is instead formulated in terms of a set of three age-dependent PDEs for the population density, irreversible mass, and reversible mass.

Population with metamorphosis

The functions $n_1(t,a)$, $x_1(t,a)$, and $y_1(t,a)$ describe the density, irreversible mass, and reversible mass of individuals before metamorphosis with age a at time t, respectively. These functions are defined over the age interval $[0,A_\text{J}]$, where A_J equals the age at which individuals reach the body mass at metamorphosis. The age at metamorphosis, A_J, is defined by the condition $(1+q_\text{J})x_1(t,A_\text{J}) = w_\text{J}$. The density, irreversible mass, and reversible mass of individuals with age a at time t are after metamorphosis described by the functions $n_2(t,a)$, $x_2(t,a)$ and $y_2(t,a)$, respectively. These densities functions are defined over the age interval $[A_\text{J},\infty]$.

The dynamics of the density of individuals with age a before metamorphosis are described by

$$\begin{aligned}
&\frac{\partial n_1(t,a)}{\partial t} + \frac{\partial n_1(t,a)}{\partial a} = -\mu(w) n_1(t,a),\\
&n_1(t,0) = \int_{A_\text{A}(t)}^{\infty} b(x_2(t,a),y_2(t,a),X_1,X_2) n_2(t,a)\mathrm{d}a.
\end{aligned} \tag{S1.11}$$

In this equation $A_\text{A}(t)$ equals the age at maturation, defined by the condition $(1+q_\text{J})x_2(t,A_\text{A}(t)) = w_\text{A}$.

The dynamics of the irreversible and reversible mass before metamorphosis are described by

$$\begin{aligned}
&\frac{\partial x_1(t,a)}{\partial t} + \frac{\partial x_1(t,a)}{\partial a} = \kappa_\text{I}(x_1(t,a),y_1(t,a)) E_\text{g}(x_1(t,a),y_1(t,a),X_1,X_2),\\
&x_1(t,0) = x_\text{b},\\
&\frac{\partial y_1(t,a)}{\partial t} + \frac{\partial y_1(t,a)}{\partial a} = \kappa_\text{R}(x_1(t,a),y_1(t,a)) E_\text{g}(x_1(t,a),y_1(t,a),X_1,X_2),\\
&y_1(t,0) = q_\text{J} x_\text{b}.
\end{aligned} \tag{S1.12}$$

During metamorphosis, individuals die with a probability of $\rho\theta$. The dynamics of the density of individuals with age a after metamorphosis ($a > A_\text{J}(t)$) are therefore described by

$$\begin{aligned}
&\frac{\partial n_2(t,a)}{\partial t} + \frac{\partial n_2(t,a)}{\partial a} = -\mu(w) n_2(t,a),\\
&n_2(t,A_\text{J}(t)) = (1-\rho\theta) n_1(t,A_\text{J}(t)).
\end{aligned} \tag{S1.13}$$

The dynamics of the irreversible mass after metamorphosis are described by

$$\frac{\partial x_2(t,a)}{\partial t}+\frac{\partial x_2(t,a)}{\partial a}=\kappa_{\mathrm{I}}(x_2(t,a),y_2(t,a))E_{\mathrm{g}}(x_2(t,a),y_2(t,a),X_1,X_2),$$
$$x_2(t,A_{\mathrm{J}}(t))=x_1(t,A_{\mathrm{J}}(t)) \tag{S1.14}$$

Since individuals lose an amount $\theta(x_{\mathrm{J}}-x_{\mathrm{b}})(q_{\mathrm{J}}-q_{\mathrm{m}})$ of their reversible body mass during metamorphosis, the dynamics of the reversible mass following metamorphosis are described by

$$\frac{\partial y_2(t,a)}{\partial t}+\frac{\partial y_2(t,a)}{\partial a}=\kappa_{\mathrm{R}}(x_2(t,a),y_2(t,a))E_{\mathrm{g}}(x_2(t,a),y_2(t,a),X_1,X_2),$$
$$y_2(t,A_{\mathrm{J}}(t))=y_1(t,A_{\mathrm{J}}(t))-\theta(x_{\mathrm{J}}-x_{\mathrm{b}})(q_{\mathrm{J}}-q_{\mathrm{m}}). \tag{S1.15}$$

In a metamorphosing population, the dynamics of the resources are given by

$$\begin{aligned}\frac{\mathrm{d}X_1}{\mathrm{d}t}&=\delta(X_{1,\max}-X_1)-\int_0^{A_{\mathrm{J}}(t)}I_1((1+q_{\mathrm{J}})x_1(t,a),X_1,X_2)n_1(t,a)\mathrm{d}a\\&\quad-\int_{A_{\mathrm{J}}(t)}^{\infty}I_1((1+q_{\mathrm{J}})x_2(t,a),X_1,X_2)n_2(t,a)\mathrm{d}a,\\\frac{\mathrm{d}X_2}{\mathrm{d}t}&=\delta(X_{2,\max}-X_2)-\int_0^{A_{\mathrm{J}}(t)}I_2((1+q_{\mathrm{J}})x_1(t,a),X_1,X_2)n_1(t,a)\mathrm{d}a\\&\quad-\int_{A_{\mathrm{J}}(t)}^{\infty}I_2((1+q_{\mathrm{J}})x_2(t,a),X_1,X_2)n_2(t,a)\mathrm{d}a.\end{aligned} \tag{S1.16}$$

Population with direct development

In a population with direct development, individuals do not undergo metamorphosis. The population is in this case fully described by the dynamics before metamorphosis. The functions $n_1(t,a)$, $x_1(t,a)$ and $y_1(t,a)$ are then defined over the age interval $[0,\infty]$ The dynamics of the density of individuals with age a are described by

$$\frac{\partial n_1(t,a)}{\partial t}+\frac{\partial n_1(t,a)}{\partial a}=-\mu(w)n_1(t,a),$$
$$n_1(t,0)=\int_{A_{\mathrm{A}}(t)}^{\infty}b(x_1(t,a),y_1(t,a),X_1,X_2)n_1(t,a)\mathrm{d}a. \tag{S1.17}$$

The dynamics of the irreversible mass are described by

$$\frac{\partial x_1(t,a)}{\partial t} + \frac{\partial x_1(t,a)}{\partial a} = \kappa_{\mathrm{I}}(x_1(t,a), y_1(t,a)) E_{\mathrm{g}}(x_1(t,a), y_1(t,a), X_1, X_2),$$
$$x_1(t,0) = x_{\mathrm{b}}. \tag{S1.18}$$

Finally, the dynamics of the reversible mass are described by

$$\frac{\partial y_1(t,a)}{\partial t} + \frac{\partial y_1(t,a)}{\partial a} = \kappa_{\mathrm{R}}(x_1(t,a), y_1(t,a)) E_{\mathrm{g}}(x_1(t,a), y_1(t,a), X_1, X_2),$$
$$y_1(t,0) = q_{\mathrm{J}} x_{\mathrm{b}}. \tag{S1.19}$$

In a population with direct development, the dynamics of the resources are given by

$$\frac{\mathrm{d}X_1}{\mathrm{d}t} = \delta(X_{1,\max} - X_1) - \int_0^\infty I_1((1+q_{\mathrm{J}})x_1(t,a), X_1, X_2) n_1(t,a) \mathrm{d}a,$$
$$\frac{\mathrm{d}X_2}{\mathrm{d}t} = \delta(X_{2,\max} - X_2) - \int_0^\infty I_2((1+q_{\mathrm{J}})x_1(t,a), X_1, X_2) n_1(t,a) \mathrm{d}a. \tag{S1.20}$$

Table S1.2: Standard parameters of the fat-reserves model

Parameter	Description	Default Value	Unit
δ	Food source turnover rate	0.1	day^{-1}
$X_{1,\mathrm{max}}$	Maximum biomass density of primary food source	variable	mg L^{-1}
$X_{2,\mathrm{max}}$	Maximum biomass density of secondary food source	variable	mg L^{-1}
w_A	Standardised body mass at maturation	8.71	g
w_0	Standardised body mass at which maximum attack rate is attained on primary food source	17.42	g
α	Exponent in attack-rate functions	0.93	-
ζ_1	Constant in handling-time function	0.00036 †	day mg^{-1}
ζ_2	Constant in handling-time function	0.00745 †	day mg^{-1}g$^{\zeta_3}$
ζ_3	Slope of decrease in handling time at small consumer sizes	0.68	-
ζ_4	Slope of increase in handling time at large consumer sizes	1.15 10^{-3}	g^{-1}
p_1	Metabolic constant	0.033	g$^{1-p_2}$day^{-1}
p_2	Metabolic exponent	0.77	–
k_e	Metabolic conversion factor	0.00061†	–
q_J	Constant determining maximum reversible body mass	0.742	–
q_A	Constant in adult allocation function	1	–
η	Gonad-offspring conversion factor	0.5	-
μ_b	Background mortality rate	0.01	day^{-1}

† These values are the original values from Persson et al. (1998) divided by $1.1 \cdot 10^{-2}$ (the weight of a prey individual) to express prey densities in milligram L^{-1} instead of individuals L^{-1}.

Table S1.3: Parameters related to specialisation and metamorphosis in the fat-reserves model

Parameter	Description	Default Value	Unit
A_{max}	Maximum value of the attack rate constants A_1 and A_2	$1\ 10^5$	L day^{-1}
A_{min}	Minimum value of the attack rate constants A_1 and A_2	$1\ 10^4$	L day^{-1}
w_{min}	Standardised body mass at which the secondary food source becomes available	0.1742	g
ς	Constant in habitat-switching rate	100	day mg^{-1}
q_{m}	Ratio of reversible to irreversible body mass immediately after full metamorphosis	0.2	–
ρ	Probability to die during full metamorphosis	0.5	-

Table S1.4: Functions of the fat-reserves model

Function	Equation
Maximum attainable reversible mass	$y_{\max} = q_J x$
Standardised body mass	$w = x + y_{\max}$
Relation between morphology of larvae, juveniles, and adults	$\psi_A = \psi_J = \min(1, \psi_L + \theta)$
Maximum attack rate on primary food source	$A_{1,i} = (1 - \psi_i)(A_{\max} - A_{\min}) + A_{\min}$
Maximum attack rate on secondary food source	$A_{2,i} = \psi_i(A_{\max} - A_{\min}) + A_{\min}$
Attack rate on primary food source	$a_1(w) = A_{1,i}[\frac{w}{w_0}\exp(1 - \frac{w}{w_0})]^{\alpha}$
Attack rate on secondary food source	$a_2(w) = \begin{cases} 0 & x < x_{\min} \\ A_{2,i}[\frac{w-w_{\min}}{w_0}\exp(1 - \frac{w-w_{\min}}{w_0})]^{\alpha} & \text{otherwise.} \end{cases}$
Handling time	$h(w) = \zeta_1 + \zeta_2 w^{-\zeta_3} e^{\zeta_4 w}$
Preference for primary food source	$\phi(w, X_1, X_2) = \frac{1}{1+e^{\varsigma(a_2(w)X_2 - a_1(w)X_1)}}$
Primary food intake	$I_1(w, X_1, X_2) = \frac{\phi(w,X_1,X_2)a_1(w)X_1}{1+h(w)\{\phi(w,X_1,X_2)a_1(w)X_1+[1-\phi(w,X_1,X_2)]a_2(w)X_2\}}$
Secondary food intake	$I_2(w, X_1, X_2) = \frac{[1-\phi(w,X_1,X_2)]a_2(w)X_2}{1+h(w)\{\phi(w,X_1,X_2)a_1(w)X_1+[1-\phi(w,X_1,X_2)]a_2(w)X_2\}}$
Total food intake	$I(w, X_1, X_2) = I_1(w, X_1, X_2) + I_2(w, X_1, X_2)$
Maintenance requirements	$E_m(x, y) = p_1(x + y)^{p_2}$
Net energy production	$E_g(x, y, X_1, X_2) = k_e I(w, X_1, X_2) - E_m(x, y)$
Fraction of net production allocated to growth in irreversible mass	$\kappa_I(x, y) = \begin{cases} \kappa_J(x, y) = \frac{y}{(1+q_J)q_J x} & \text{if } (1 + q_J)x < w_A \\ \kappa_A(x, y) = \frac{y}{(1+q_A)q_A x} & \text{otherwise} \end{cases}$
Fraction of net production allocated to growth in reversible mass	$\kappa_R(x, y) = \begin{cases} 1 - \kappa_J(x, y) & \text{if } (1 + q_J)x < w_A \\ 1 - \kappa_A(x, y) & \text{if } y < q_J \text{ and } (1 + q_J)x \geq w_A \\ [1 - \kappa_J(x, y)]\frac{\kappa_A(x,y)}{\kappa_J(x,y)} & \text{otherwise} \end{cases}$
Fecundity of adults in metamorphosing species	$b(x, y, X_1, X_2) = \begin{cases} 0 & \text{if } y < q_J \\ [1 - \frac{\kappa_A(x,y)}{\kappa_J(x,y)}]\frac{\eta E_g(x,y,X_1,X_2)}{(1+q_J)x_b} & \text{otherwise} \end{cases}$
Fecundity of adults in species with direct development	$b(x, y, X_1, X_2) = \begin{cases} 0 & \text{if } y < q_J \\ [1 - \frac{\kappa_A(x,y)}{\kappa_J(x,y)}](1 - \rho\theta)\frac{\eta E_g(x,y,X_1,X_2)}{(1+q_J)x_b} & \text{otherwise} \end{cases}$
Amount of reversible mass lost during metamorphosis	$\theta(x_J - x_b)(q_J - q_m)$
Probability to die during metamorphosis	$\rho\theta$

The index i indicates a certain life stage (larvae, juveniles, or adults; $i =$ L, J, or A).

Methods S2

Description of the generic size-structured population model

As in the fat-reserves model, we model a primary and secondary food source, with biomass densities X_1 and X_2 respectively. The food sources follow semi-chemostat dynamics with turnover rate δ and reach densities of $X_{1,\max}$ and $X_{2,\max}$ in the absence of the consumer population.

In this generic size-structured model, we do not distinguish between irreversible and reversible body mass of consumers; individuals are only characterised by their total body size s. Larvae (L) are born with a body size of s_{b}, get access to the secondary food source at a body size of $s_{\min}$, undergo metamorphosis and become juveniles (J) at a body size of s_{j}, and become mature adults (A) when reaching a body size of s_{m}. Rates of food intake are linearly related to individual body size. We assume a Holling-type-2 functional response; therefore, the size-specific food intake of individuals with body size s can be written as

$$I(s,X_1,X_2) = \begin{cases} \frac{a_{1,\mathrm{L}}X_1}{1+ha_{1,\mathrm{L}}X_1} & s < s_{\min} \text{ and } i = \mathrm{L} \\ \frac{\phi a_{1,i}X_1+(1-\phi)a_{2,i}X_2}{1+h(\phi a_{1,i}X_1+(1-\phi)a_{2,i}X_2)} & \text{otherwise.} \end{cases} \tag{S2.1}$$

In this equation, the parameters $a_{1,i}$ and $a_{2,i}$ are the size-specific attack rates of individuals in a certain life stage (i = L, J, or A) on the primary and secondary food source, respectively. The parameter h is the size-specific handling time and parameter ϕ is the relative preference of large individuals ($s > s_{\min}$) for the primary food source (see equation S1.5 in Methods S1).

Ingested food is assimilated with efficiency ε and first used to cover maintenance costs. We assume that maintenance requirements scale linearly with body size with proportionality constant T. The size-specific net biomass production of individuals is determined by the difference between food assimilation $\varepsilon I(s,X_1,X_2)$ and maintenance costs. The net biomass production per unit body size as a function of the resource densities then equals

$$\nu(s,X_1,X_2) = \varepsilon I(s,X_1,X_2) - T. \tag{S2.2}$$

Immature individuals use their net biomass production to grow in body size, while mature individuals ($s = s_{\mathrm{m}}$) do not grow and use all their net energy production for reproduction. The growth rate of immature individuals ($s < s_{\mathrm{m}}$) equals

$$g(s,X_1,X_2) = \nu(s,X_1,X_2)s, \tag{S2.3}$$

and adult reproduction equals

$$b(s,X_1,X_2) = \nu(s,X_1,X_2)s_{\mathrm{m}}/s_{\mathrm{b}}. \tag{S2.4}$$

All individuals experience a daily background mortality rate of μ_{b}.

We assume again a trade-off between the attack rates on the primary and secondary food source such that the attack rates in a certain life stage are

$$a_{1,i} = (1-\psi_i)(A_{\max} - A_{\min}) + A_{\min}, \tag{S2.5a}$$

$$a_{2,i} = \psi_i(A_{\max} - A_{\min}) + A_{\min}. \tag{S2.5b}$$

In these equations, parameter ψ_i indicates the relative degree of specialisation of a life stage ($i =$ L, J, or A) on the secondary food source. Metamorphosis decouples the different life stages such that

$$\psi_{\mathrm{A}} = \psi_{\mathrm{J}} = \min(1, \psi_{\mathrm{L}} + \theta), \tag{S2.6}$$

where parameter θ is the extent of the metamorphosis. As in the main model, we assume that individuals have a probability of $\rho\theta$ to die during metamorphosis. Individuals lose $\theta(1-q_{\mathrm{s}})(s_{\mathrm{j}} - s_{\mathrm{b}})$ of their body size when they undergo metamorphosis ($s = s_{\mathrm{j}}$). Note that it is possible that individuals become, after metamorphosis, smaller than $s_{\min}$, the minimum size needed to feed on the secondary food source. For simplicity, however, we assume that individuals can always feed on the secondary food source after metamorphosis, independent of their body mass.

Maintenance rate, attack rate, and maximum ingestion rate (which is the inverse of the handling time), are all size-specific. Default values of these parameters (see table S2.1) are derived from the scaling relations of these constants with the adult body weight s_{m} as presented by de Roos and Persson 2013. For the adult body size, we choose a value of 0.1 mg. We vary the body size at which the secondary food source becomes available ($s_{\min}$). We choose a value of 0.5 for parameter ρ, the probability to die during full metamorphosis. For parameter q_{s} we choose a value of 0.6. The model-specific file needed for the analysis with the PSPMpackage together with an R script that executes all the calculations made in this appendix have been made available in the Dryad data repository.

We used the framework of adaptive dynamics to study the evolution of four traits; the extent of metamorphosis θ, specialisation parameter ψ_{L}, the body size at birth s_{b} and the body size at which metamorphosis takes place s_{j}. We used the canonical equation of adaptive dynamics to study to which values the traits evolve for a certain set of parameters. For all parameters investigated, we find that the four evolving traits always keep changing over evolutionary time. However, for a fixed set of parameters, the change in the trait values becomes over evolutionary time extremely small (e.g., for the body size at birth the change in the trait value is of the order of 1 x 10^{-5} mg) and biologically no longer relevant. We therefore show the values to which the traits converge, even though the strategies are not continuously stable strategies.

Table S2.1: Parameters of the generic size-structured model

Parameter	Description	Default value	Unit
δ	Food-source turnover rate	0.1	day^{-1}
$X_{1,\max}$	Maximum biomass density of primary food source	-	mg L^{-1}
$X_{2,\max}$	Maximum biomass density of secondary food source	-	mg L^{-1}
σ	Constant in habitat-switching rate	100	d
$A_{\max}$	Maximum size-specific attack rate	0.6	L mg^{-1} day^{-1}
$A_{\min}$	Minimum size-specific attack rate	0.06	L mg^{-1} day^{-1}
ε	Conversion efficiency	0.5	-
h	size-specific handling time	1	day
T	size-specific maintenance rate	0.1	day^{-1}
s_{m}	Adult weight	0.1	mg
$s_{\min}$	Body size at which secondary food source becomes available	-	mg
μ_{b}	Mortality rate	0.02 day^{-1}	
ρ	Probability to die during full metamorphosis ($\theta = 1$)	0.5	-
q_{s}	Fraction of original body size that is left after full metamorphosis ($\theta = 1$)	0.6	-
ψ_{L}^{*}	Degree of specialisation of larvae on the secondary food source	From 0 to 1	-
θ^{*}	Extent of metamorphosis	From 0 to 1	-
s_{b}^{*}	Newborn weight	From 5x10^{-6} to s_{m}	mg
s_{j}^{*}	Weight at metamorphosis	From 5x10^{-6} to s_{m}	mg

* Parameter can change due to evolution

Table S3

Table S3.1: Differences and similarities between the two models

	Fat-reserves model	Generic model
	**Differences**	
Growth after maturation	Continues	Stops
Fecundity	Increases with body mass	Same for all adults
Body mass	Reversible and irreversible structure	No differentiation
Allocation to fat	Depending on body mass and ratio x and y	-
Attack rates	Hump-shaped functions of body mass	Linear increase with body mass
Energetic costs of metamorphosis	Decrease of fat reserves	Decrease of total body mass
Parameters	Roach	Invertebrate species
	**Similarities**	
Food source dynamics	Semi-chemostat dynamics	
Availability of the secondary food source	Large individuals only	
Relative preference for food sources	Dependent on food source densities and specialisation	
Trade-off between attack rates	Linear trade-off	
Food intake	Holling-type-2 functional response	
Advantage of metamorphosis	Decoupling of the life-stages	
Mortality risk of metamorphosis	$\rho\theta$	
Evolving traits	Extent of metamorphosis, juvenile specialisation, size at birth, size at metamorphosis	
Background mortality	Independent of body mass and habitat (except in appendix C)	

Methods S4

Description of the IBM

To study the evolutionary response of a metamorphosing population to deteriorating food conditions, we used the framework of adaptive dynamics. One of the main assumptions of this framework, is that ecological processes take place on a much faster timescale than evolutionary process (Geritz et al. 1998). We furthermore assumed an infinite population size and ignored stochastic processes. Here, we use an individual based model (IBM) to study how relaxing these assumptions affects our results. The IBM is based on the same life history as the deterministic model of the main text, described in Methods S1. In the main text, the dynamics of the system involve densities (biomass per litre). In the IBM, however, individual consumers are discrete entities, such that birth and death events can only occur as discrete, stochastic, events. We therefore also have to specify the size of the system, which might affect the population dynamics (e.g., Nisbet et al. 2016) and therefore the evolutionary outcome (e.g., Claessen et al. 2007, 2008). All functions of the model are described in Methods S1. Below, we describe the update rules for the IBM. We implemented the model in C++, the code has been made available in the Dryad data repository.

We assume that the supply rate of the primary food source decreases linearly over time with an amount of ξ per day. The higher parameter ξ, the faster the primary food source deteriorates. For the two food sources, the change ΔX_1 and ΔX_2 in a time step Δt equals

$$\begin{aligned}\Delta X_1 &= [\delta(X_{1,\max} - \xi t - X_1) - \sum(I_1(X_1,X_2,w))/s]\Delta t,\\ \Delta X_2 &= [\delta(X_{2,\max} - X_2) - \sum(I_2(X_1,X_2,w))/s]\Delta t,\end{aligned} \tag{S4.1}$$

where s is the size of the system in litres and $\sum(I_i(X_1,X_2,w))$ is the sum of the intake of food source i over all individual consumers. The food intake of food source X_1 and X_2 of an individual with body mass w is given by

$$\begin{aligned}I_1(X_1,X_2,w) &= \frac{\phi(w,X_1,X_2)a_1(w)X_1}{1+h(w)\{\phi(w,X_1,X_2)a_1(w)X_1+[1-\phi(w,X_1,X_2)]a_2(w)X_2\}},\\ I_2(X_1,X_2,w) &= \frac{[1-\phi(w,X_1,X_2)]a_2(w)X_2}{1+h(w)\{\phi(w,X_1,X_2)a_1(w)X_1+[1-\phi(w,X_1,X_2)]a_2(w)X_2\}}.\end{aligned} \tag{S4.2}$$

The total food intake rate by an individual with body mass w equals

$$I(X_1,X_2,w) = \frac{\phi(w,X_1,X_2)a_1(w)X_1+[1-\phi(w,X_1,X_2)]a_2(w)X_2}{1+h(w)\{\phi(w,X_1,X_2)a_1(w)X_1+[1-\phi(w,X_1,X_2)]a_2(w)X_2\}}, \tag{S4.3}$$

see also equation S1.4 in Methods S1.

In case the total energy intake of an individual ($\kappa_e(I(X_1,X_2,w))\Delta t$) is larger than its mainte-

nance costs ($E_m(x,y)\Delta t$, equation S1.6), its growth in irreversible mass in a time step equals

$$\kappa_J(x,y)E_g(X_1,X_2,w)\Delta t \tag{S4.4}$$

for larvae and juveniles ($w < w_A$) and

$$\kappa_A(x,y)E_g(X_1,X_2,w)\Delta t \tag{S4.5}$$

for adults. The growth in reversible mass in a time step equals

$$(1-\kappa_J(x,y))E_g(X_1,X_2,w)\Delta t \tag{S4.6}$$

for larvae and juveniles ($w < w_A$) and

$$\kappa_R(x,y)E_g(X_1,X_2,w)\Delta t \tag{S4.7}$$

for adults. The remainder fraction of the adult net-biomass production is invested in their reproduction buffer. Mature individuals with enough energy to reproduce, produce as many discrete offspring as they have energy for in their reproduction buffer. The costs of producing a single offspring equals w_b/η in case metamorphosis takes place after birth, and $w_b)(\eta(1-\rho\theta))$ in case metamorphosis takes place before birth (see equations S1.9 and S1.10 in Methods S1).

Newborn individuals have a mutation probability of υ for each of the four evolving traits (ψ_L, θ, x_b, and x_J). In case a mutation occurs in one of the evolving traits, the offspring trait equals the trait of the parent $+p$, with p normally distributed with a mean of zero and standard deviation σ.

In contrast to the main model, we allow starvation to occur when total energy intake is insufficient to cover maintenance costs. Initially, individuals will use their reserves to cover maintenance costs, but will suffer from starvation mortality when $y \leq q_s x$. The probability to die of starvation within a time step Δt is $\mu_s(x,y)\Delta t$, which increases with decreasing reserves. The function $\mu_s(x,y)$ equals

$$\mu_s(x,y) \begin{cases} \lambda(q_s\frac{x}{y}-1)\Delta t & y \leq q_s x \\ 0 & y > q_s x, \end{cases} \tag{S4.8}$$

where λ is a proportionality constant. In addition to starvation mortality, individuals have a probability to die from background mortality within a time step Δt equal to $\mu_b \Delta t$.

For each time step Δt, we first calculate the changes in state variables and update them. Individuals that reach irreversible body mass $x \geq x_J$ in this time step, metamorphose, lose $\theta(x_J - x_b)(q_J - q_m)$ of their reversible body mass, and die with probability $\rho\theta$. Next, we remove consumers that have died (due to starvation, background mortality, or metamorphosis) from the population. Lastly, adult individuals reproduce new offspring, which are added to the population.

To determine which individual dies in a certain time step Δt, we draw for each individual a random number from a uniform distribution on the interval $[0,1)$. If this number is smaller than the mortality probability for this individual, it dies. We use a similar approach to determine which offspring mutates in which trait. For each newborn, we draw for each of the four evolving trait a random number from a uniform distribution on the interval $[0,1)$. The newborn will get a mutation

in a certain trait in case this random number is smaller than the mutation probability υ.

We use a time step of $\Delta t = 0.1$ for all simulations. We run simulations for two values of $\delta X_{2,\max}$; a low value for which the population goes extinct in the deterministic model, and a high value for which direct development evolves in the deterministic model. For the mutation probability υ, standard deviation of the normal distribution σ, system volume s, and speed of habitat deterioration ξ, we use two different values each (table B1). Each combination was simulated 4 times, resulting in a total of 64 runs for each $\delta X_{2,\max}$ value. Other parameters are as in tables 1, S1.2, and S1.3.

We start each simulation with a high supply rate of the primary food source, $\delta X_{1,\max} = 0.0033$ mg l^{-1}. The population is initially monomorphic in the four evolving traits. The initial trait values correspond to the trait values of the ESS of the deterministic model. We initialise the consumer population with 100 newborn individuals and run the simulations for 10.000 days in the absence of evolution (mutation probability $\upsilon = 0$), and no habitat deterioration ($\xi = 0$). After this initialisation, we start the evolutionary simulations as described above. The simulations run until the consumer population is extinct or until direct development has evolved. To see what happens when the supply rate of the primary food source reaches the level where direct development can evolve, we also study the evolutionary dynamics while keeping the supply rate at this constant value. This allows us to study if in the IBM direct development evolves in a similar manner as in the deterministic model.

Supplementary material S5

Population level densities

In figures S5.1 and S5.2 we show how the food source densities (panel A and B), population densities (panel C), and age at metamorphosis (panel D) change as a function of the supply rate of the primary food source. We show these values for one situation where a metamorphosing population evolves direct development (figure S5.1), and for one situation where metamorphosis is an evolutionary trap (figure S5.2). To highlight the feedback between evolution and ecology, we show these values for two populations, a population that is allowed to evolve (solid lines) and a population that is not allowed to evolve (dashed lines). In the absence of evolution, both of the non-evolving populations go extinct for low supply rates of the primary food source.

Decreasing the productivity of the primary food source results in stronger competition for this food source. In the absence of evolution, this leads to a decrease of the density of the primary food source (dashed lines in figures S5.1A and S5.2A) and an increase in the age at metamorphosis (dashed lines in figures S5.1D and S5.2D). Fewer individuals will therefore metamorphose (dashed orange lines in figures S5.1C and S5.2C), which results in an increase in the density of the secondary food source (dashed lines in figures S5.1B and S5.2B).

The evolutionary response to decreasing supply rates of the primary food source is a larger body mass at birth in combination with a smaller body mass at metamorphosis (figure 2 and 4 in the main text). Due to this evolutionary response, individuals metamorphose at an earlier age (solid lines in figures S5.1D and S5.2D), even though the density of the primary food source decreases (solid lines in figures S5.1A and S5.2A). Due to the earlier maturation, the number of larvae decreases faster in the evolving populations compared to the non-evolving populations (green lines in figures S5.1C and S5.2C). This early metamorphosis results in relatively more metamorphosed individuals in the evolving population compared to the non-evolving population (orange lines in figures S5.1C and S5.2C). This higher density of metamorphosed individuals increases the competition for the secondary food source, resulting in a lower density of this food (figures S5.1B and S5.2B).

Note that in the situation where a metamorphosing population goes extinct, the density of the primary food source is at some point higher in the absence of evolution compared to in the presence of evolution (figure S5.2A). The reason for this is that in the absence of evolution, the population becomes very small for low supply rates (figure S5.2C). The non-evolving population therefore hardly impacts the food densities, which leads to relatively higher food levels.

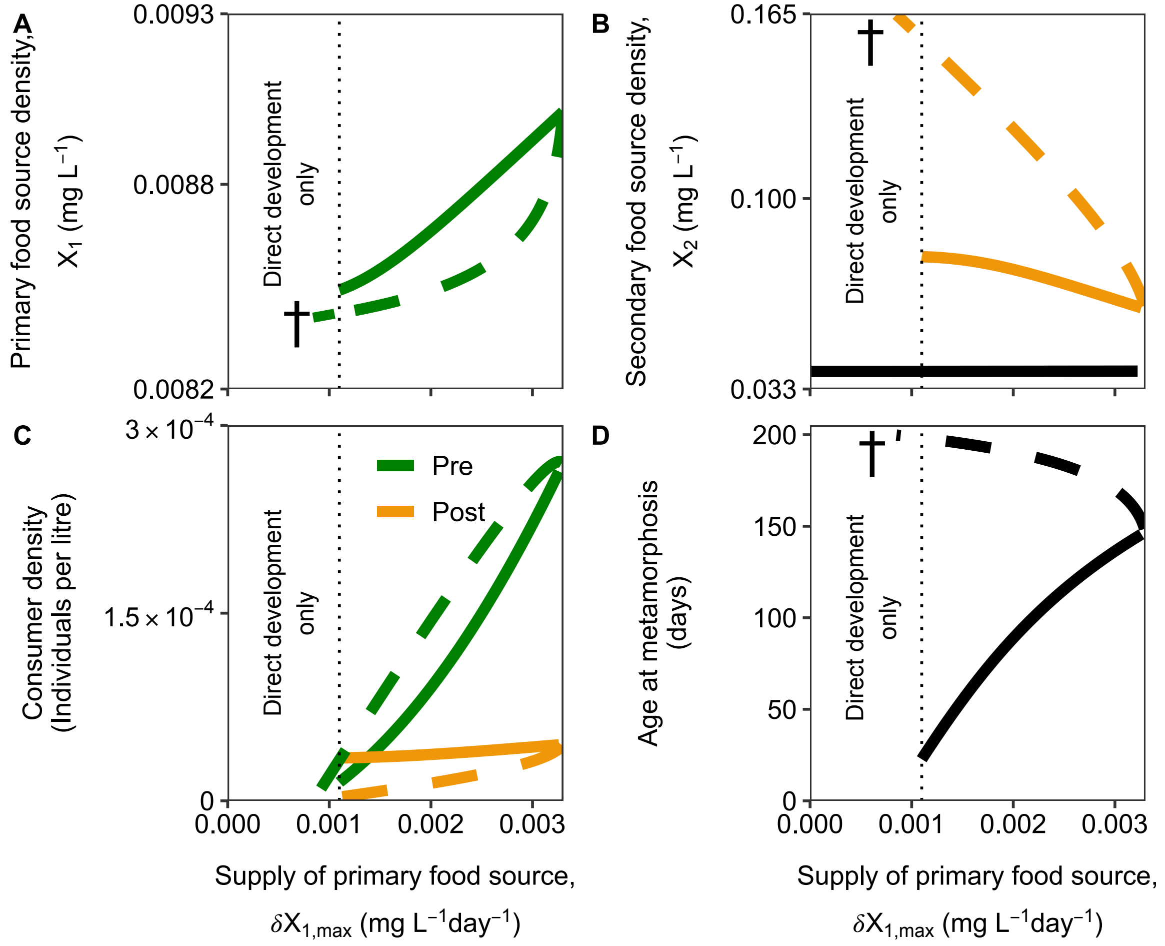

Figure S5.1: Density of the primary (panel A) and secondary (panel B) food source (mg L^{-1}), (C) population density (individuals per litre), and (D) age at metamorphosis (days) as a function of the supply rate of the primary food source (mg L^{-1}day^{-1}) in the presence (solid lines) and absence (dashed lines) of evolution for a species with metamorphosis. The black line in panel B represents the density of the secondary food source for a population with direct developers. The dark-green line in panel (C) represents the density of individuals before metamorphosis (with a body mass $w < w_J$), the orange line represents the density of individuals after metamorphosis. The vertical dotted line in all panels indicate at which value of the supply rate the population evolves direct development. In the absence of evolution, the population will go extinct for low supply rates of the primary food source (indicated with a dagger). The trait values for the population without evolution are $\theta = 0.88$, $\psi_L = 0$, $w_J = 0.202$, and $w_b = 0.037$, which are the trait values in the ESS for a supply rate of the primary food source of 0.0033 mg L^{-1}day^{-1}.The supply rate of the secondary food source equals $\delta X_{2,max} = 0.0165$ mg L^{-1}day^{-1}. Other parameter values are as shown in tables S1.2 and S1.3.

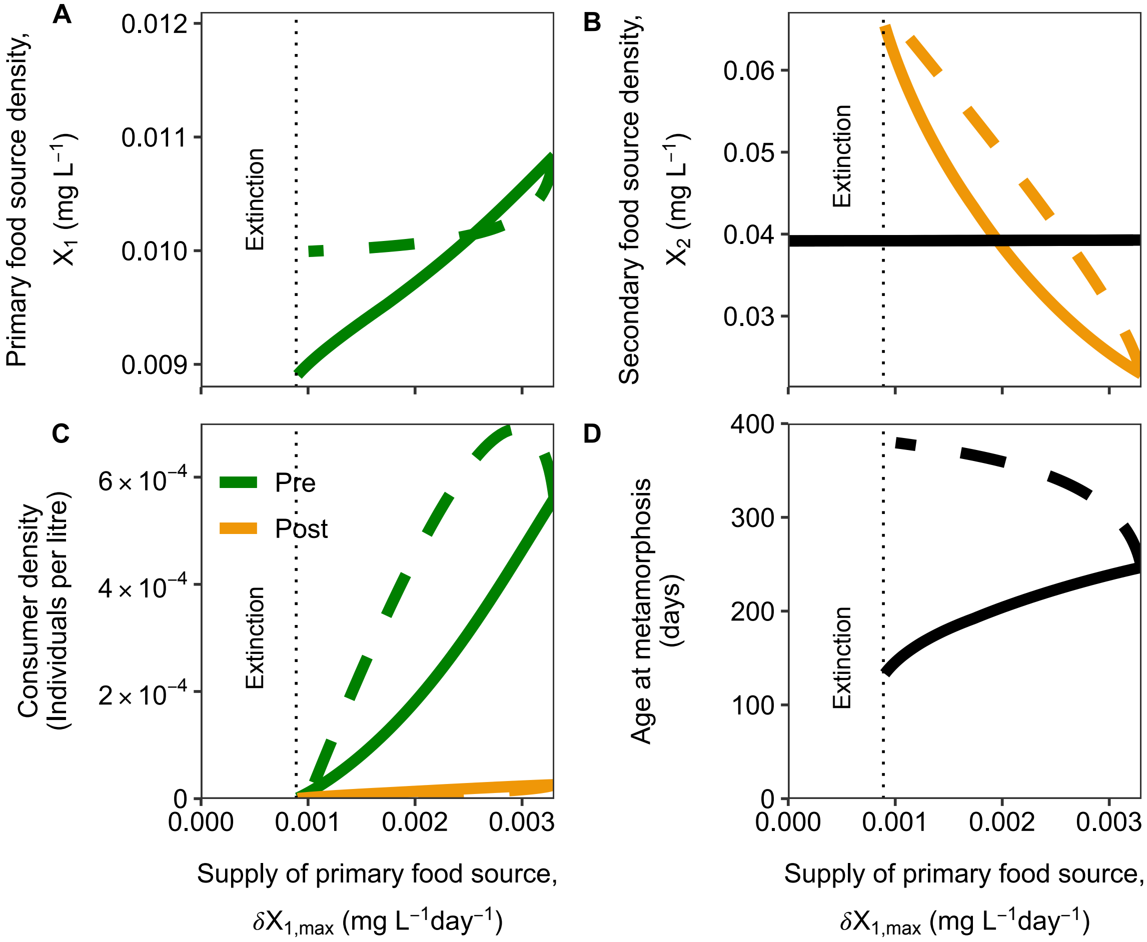

Figure S5.2: Density of the primary (panel A) and secondary (panel B) food source (mg L^{-1}), (C) population density (individuals per litre), and (D) age at metamorphosis (days) as a function of the supply rate of the primary food source (mg L^{-1}day^{-1}) in the presence (solid lines) and absence (dashed lines) of evolution for a species with metamorphosis. The black line in panel B represents the density of the secondary food source for a population with direct developers. The dark-green line in panel (C) represents the density of individuals before metamorphosis (with a body mass $w < w_J$), the orange line represents the density of individuals after metamorphosis. The vertical dotted line in all panels indicate at which value of the supply rate the population that is allowed to evolve goes extinct. In the absence of evolution, the population will go extinct at a slightly higher supply rate. The trait values for the population without evolution are $\theta = 1$, $\psi_L = 0$, $w_J = 0.31$, and $w_b = 0.0042$, which are the trait values in the ESS for a supply rate of the primary food source of 0.0033 mg L^{-1}day^{-1}. The supply rate of the secondary food source equals $\delta X_{2,max} = 0.0066$ mg L^{-1}day^{-1}. Other parameter values are as shown in tables S1.2 and S1.3.
